# Early Blood Pressure Targets in Acute Spinal Cord Injury: A Randomized Clinical Trial

**DOI:** 10.1001/jamanetworkopen.2025.25364

**Published:** 2025-09-18

**Authors:** Ruba Sajdeya, N. David Yanez, Michael Kampp, Michael D. Goodman, David Zonies, Brandon Togioka, Andrew Nunn, Robert D. Winfield, Niels D. Martin, Anirudh Kohli, Toan T. Huynh, David O. Okonkwo, Roy A. Poblete, Emily J. Gilmore, Randall M. Chesnut, Aaron E. Bunnell, Tetsu Ohnuma, Mona Hashemaghaie, Miriam M. Treggiari

**Affiliations:** 1 Department of Anesthesiology, Duke University School of Medicine, Durham, North Carolina; 2 Department of Biostatistics and Bioinformatics, Duke University School of Medicine, Durham, North Carolina; 3 Division of Neurocritical Care and Emergency Neurology, Yale University School of Medicine, New Haven, Connecticut; 4 Department of Surgery, University of Cincinnati, Cincinnati, Ohio; 5 Department of Surgery, Oregon Health and Science University, Portland, Oregon; 6 Department of General Surgery, Wake Forest University School of Medicine, Winston-Salem, North Carolina; 7 Division of Acute Care Surgery, University of Kansas Medical Center, Kansas City, Kansas; 8 Department of Surgery, University of Pennsylvania Health System, Philadelphia, Pennsylvania; 9 Department of Surgery, Thomas Jefferson University, Philadelphia, Pennsylvania; 10 Division of Trauma, Surgical Critical Care and Acute Care Surgery, Atrium Health Carolinas Medical Center, Charlotte, North Carolina; 11 Division of Neurological Surgery, University of Pittsburgh Medical Center, Pittsburgh, Pennsylvania; 12 Department of Neurology, University of Southern California, Los Angeles; 13 Department of Neurological Surgery, University of Washington, Seattle

## Abstract

**Importance:**

Early blood pressure management is central to neurologic resuscitation of spinal cord injury; however, the role of augmented blood pressure is unclear.

**Objective:**

To compare the efficacy and safety of augmented vs conventional blood pressure on 6-month neurologic outcomes after acute spinal cord injury.

**Design, Setting, and Participants:**

This multicenter randomized clinical trial took place from October 3, 2017, to July 26, 2023, and assessed patients 18 years or older with spinal cord injury followed up for 6 months at 13 large US trauma centers.

**Interventions:**

Patients were equally randomized to augmented (>85-90 mm Hg) or conventional (>65-70 mm Hg) mean arterial pressure for 7 days or until intensive care unit discharge.

**Main Outcomes and Measures:**

Primary end points were change in motor and sensory American Spinal Injury Association Impairment Scale scores from baseline to 6 months. Safety end points included organ dysfunction and complications.

**Results:**

The trial randomized 92 patients (mean [SD] age, 53.78 [18.74] years; 76 [83%] male). At 6 months, 38 patients had completed follow-up and 15 had died. Among survivors, there were no mean (SD) differences in change from baseline in upper extremity motor scores (34.95 [3.25] vs 32.95 [3.65]; difference, 2.48; 95% CI, −5.93 to 10.90; *P* = .55), lower extremity motor scores (18.53 [4.62] vs 19.95 [4.59]; difference, −4.56; 95% CI, −16.11 to 7.03; *P* = .43), or total sensory scores (108.47 [12.49] vs 130.89 [14.87]; difference, −32.00; 95% CI, −65.40 to 1.40; *P* = .06) comparing the augmented and conventional groups. The augmented group had higher mean (SD) modified Sequential Organ Failure Assessment scores (excluding cardiovascular components) at day 3 (1.65 [1.79] vs 0.80 [1.10]; difference, 0.85; 95% CI, 0.23-1.47; *P* = .008) and day 6 (1.55 [1.82] vs 0.80 [1.35]; difference, 0.74; 95% CI, 0.05-1.44; *P* = .04), longer mechanical ventilatory support (9.44 [15.27] vs 3.78 [8.42] days; difference, 5.67 days; 95% CI, 0.48-10.85 days; *P* = .03), and more respiratory complications (36 [78%] vs 18 [39%]; risk difference, 40%; 95% CI, 22%-58%; *P* < .001) than the conventional group. No differences in mortality or other secondary outcomes were observed.

**Conclusions:**

Although underpowered, this randomized clinical trial of patients with spinal cord injury did not demonstrate better neurologic recovery comparing early augmented and conventional blood pressure and calls this practice into question. Further study is needed to identify groups who may benefit from augmenting blood pressure and determine potential harm mechanisms.

**Trial Registration:**

ClinicalTrials.gov Identifier: NCT02878850

## Key Points

**Question:**

Compared with conventional blood pressure, does early blood pressure augmentation improve long-term neurologic outcomes in acute spinal cord injury?

**Findings:**

This multicenter randomized clinical trial of 92 patients with spinal cord injury did not find differences in 6-month motor or sensory scores between augmented and conventional blood pressures. The augmented blood pressure group had higher respiratory complications, longer mechanical ventilatory support, and worse organ dysfunction.

**Meaning:**

These findings call into question the efficacy and safety of blood pressure augmentation due to higher complications without appreciable differences in neurologic function.

## Teaser

This randomized clinical trial of patients with spinal cord injury examines whether augmented blood pressure (mean arterial pressure >85-90 mm Hg) improves motor and sensory scores 6 months after injury.

## Introduction

Spinal cord injury (SCI) is a leading cause of long-term disability, accounting for 4.5 million years of life lived with disability worldwide in 2021.^1,2^ Although SCI may initially present with irreversible damage, adherence to treatment protocols to prevent secondary injury is associated with long-term improvement in neurologic and functional outcomes.^3,4,5,6,7^ Evidence supports preventing hypotension as a critical component of early SCI management to improve outcomes.^6,8^ The 2013 American Association of Neurological Surgeons and Congress of Neurological Surgeons SCI guideline recommends maintaining a mean arterial pressure (MAP) of 85 to 90 mm Hg for 7 days.^9^ However, this level III recommendation was based on low-quality evidence of neurologic improvement and has not been studied in randomized clinical trials. More recent guidelines recommended maintaining a MAP of 75 to 80 mm Hg for 3 to 7 days, but this was a similarly weak recommendation based on very low quality evidence.^10^

Early targeted hemodynamic management’s role in SCI treatment and its impact on long-term neurologic impairment have not been systematically investigated. Targeted blood pressure management in the initial phase of neurologic resuscitation assumes that spinal cord blood flow is pressure dependent due to disrupted autoregulation^11^ and that preservation of adequate spinal cord perfusion pressure results in clinical benefits.^12,13^ However, not only is the extent of impairment of spinal cord autoregulation unknown, but potent vasoconstrictors could potentially impair spinal cord blood flow.^14,15^ Additional concerns with augmented MAP targets include the need for invasive procedures, vasopressors, and prolonged intensive care unit (ICU) stay.^16^

To address the controversy over the efficacy and safety of blood pressure targets in acute SCI, we conducted a trial of patients with acute SCI randomized to augmented blood pressure (ABP) with a MAP goal of greater than 85 to 90 mm Hg or conventional blood pressure (CBP) with a MAP goal of greater than 65 to 70 mm Hg maintained for up to 7 days after injury. We hypothesized that APB would improve the mean change of American Spinal Injury Association (ASIA) Impairment Scale (AIS) motor and sensory scores 6 months after injury without increasing adverse events.

## Methods

### Study Design and Setting

This multicenter randomized clinical trial assessed the efficacy and safety of early blood pressure augmentation in ICU patients with acute SCI followed up for 6 months at 13 US trauma centers between October 3, 2017, and July 26, 2023. The study received ethical approval from the institutional review board at Oregon Health & Science University and from each participating site. All participants provided written informed consent. The full study protocol is available in Supplement 1. The study followed the Consolidated Standards of Reporting Trials (CONSORT) reporting guideline.

### Eligibility

Patients with acute SCI presenting to one of the participating centers were screened. Patients eligible for enrollment were 18 years or older with acute traumatic SCI involving the cervical or thoracic spine (C0-T8) and resulting in new-onset neurologic deficits with an AIS grade of A, B, or C and consistent with radiological findings. Patients were excluded if they presented with penetrating SCI, injuries at or below T9, isolated cauda equina syndrome, or severe traumatic brain injury (Glasgow Coma Scale score ≤8 with intracranial abnormalities on imaging); had preexisting motor deficits from chronic myelopathy, history of demyelinating diseases or central nervous system autoimmune disorders, any condition preventing accurate neurologic exam (eg, Alzheimer disease, stroke, degenerative conditions, cerebral tumors, or intellectual disability), decompensated congestive heart failure (New York Heart Association functional class III or IV or objective class C or D), myocardial infarction within 6 months, end-stage kidney disease, terminal diagnosis with a life expectancy less than 6 months, or suspected or confirmed pregnancy; did not speak English or Spanish; or declined informed consent.

### Randomization and Blinding

After enrollment and baseline assessment, eligible patients were equally randomized to 1 of 2 study groups: ABP (target MAP >85-90 mm Hg) or CBP (target MAP >65-70 mm Hg). Randomization was performed using a computer-based unrestricted 1:1 fair-coin design. Clinical assessors determining baseline neurologic examinations, AIS assessments, and eligibility were blinded to randomization assignment until informed consent was obtained and enrolling procedures were completed. Assessors of the 6-month follow-up were also blinded to treatment assignment. Due to requiring specific blood pressure targets, the treating team was not blinded to MAP allocation.

### Intervention

Participants were assigned to their respective blood pressure targets for up to 7 days after randomization or until ICU discharge, whichever came first. MAP targets were achieved by administering vasoactive medications, including phenylephrine, norepinephrine, vasopressin, dopamine, or a combination of these agents, per institutional standard of care titrated to the assigned blood pressure goals. Patients received fluid resuscitation to maintain normovolemia. Hemodynamic values were recorded prior to randomization and then every 4 hours for 7 days (see the trial protocol in Supplement 1).

### Study End Points

The primary end point was the change from baseline in AIS motor and sensory scores 6 months after injury. AIS scores were determined per the International Standards for Neurological Classification of SCI recommendations. Prerandomization ASIA assessments were recorded at baseline, documenting SCI severity, level, and mechanism of injury. ASIA assessment was repeated within 72 hours after injury if the study-qualifying baseline assessment was incomplete and again at 6 months. The secondary end points included pain, performance in activities of daily living and mobility, and quality of life collected at the 6-month follow-up visit via the International Spinal Cord Injury Basic Pain Data Set,^17^ the Spinal Cord Independence Measure,^18^ and the International Spinal Cord Injury Quality of Life Basic Data Set,^19^ respectively (see the trial protocol in Supplement 1).

### Safety Outcomes

Safety assessments included treatment discontinuation due to treatment-associated complications or need to change the assigned MAP goals due neurologic deterioration, daily Sequential Organ Failure Assessment (SOFA) score during treatment,^20^ and reports of adverse events and serious adverse events at 6 months. We excluded the cardiovascular points from the SOFA score to avoid penalizing the APB group for points contributed by the study intervention. Respiratory complications included acute respiratory distress syndrome, hypoxemia (ratio of Pao_2_ to fraction of inspired oxygen <200 mm Hg), pulmonary edema requiring diuretics, and pneumonia. We also collected duration of ventilatory support (invasive or noninvasive), including continuous positive airway pressure delivered through a facemask, endotracheal tube or tracheotomy duration of hospitalization, and discharge disposition.

### Statistical Analysis

To detect the primary end point of change in AIS scores at 6 months, a sample of 126 patients was needed to detect a clinically meaningful 5-point difference in neurologic recovery between the treatment groups with a power of 80%. We assumed a 10-point SD in AIS scores using a 2-sided α = .05^13^ and that the correlation between baseline AIS scores and 6-month AIS scores was modest (*r* = 0.50).

We performed a complete-case, intention-to-treat analysis to compare mean 6-month ASIA sensory and motor scores between the ABP and CBP groups using analysis of covariance, adjusting for patients’ baseline ASIA scores. We reported the estimated model regression coefficient for the treatment indicator, interpreted as the difference in the mean change from baseline in AIS sensory and motor function test scores at 6 months between the ABP and CBP treatment groups. We used robust (sandwich) SE estimates for our statistical tests and 95% CIs to account for the possibility of variance heterogeneity in the 6-month AIS scores. We visualized changes in AIS severity grade using clustered bar charts with Sankey diagrams. We performed an ad hoc sensitivity analysis to evaluate missing data resulting from patient deaths before follow-up. We jointly modeled time to death and follow-up scores 6 months after injury. We first imputed risk estimates for death using Cox proportional hazards regression. The risk estimates were incorporated into multiple imputation models for 6-month follow-up outcomes (ie, AIS scores). Predictors in the imputation models included baseline AIS scores and the treatment indicator. We generated 20 imputation datasets and combined them for these analyses using Rubin rules. Finally, we compared the multiple imputation results with our complete case analyses. Each outcome was modeled separately.

We descriptively reported baseline characteristics and secondary and safety end points in tabular format using means (SDs) or numbers (percentages), stratified by treatment group. Race and ethnicity were self-reported and categorized as follows: Hispanic, non-Hispanic Black, non-Hispanic White, and other (American Indian or Alaska Native, Asian, Native Hawaiian or Other Pacific Islander, unknown, and unspecified or not reported). We collected data on race and ethnicity to evaluate the representativeness of the study population. Two-sample *t* tests and χ^2^ statistics were used as appropriate to compare treatment groups. Missing data were reported for every variable in tabular format. All statistical tests were 2-sided, with an α = .05 for statistical significance, and performed with SAS software, version 9.4 (SAS Institute Inc) and Stata/MP software, version 18.0 (StataCorp).

## Results

**Figure 1.**
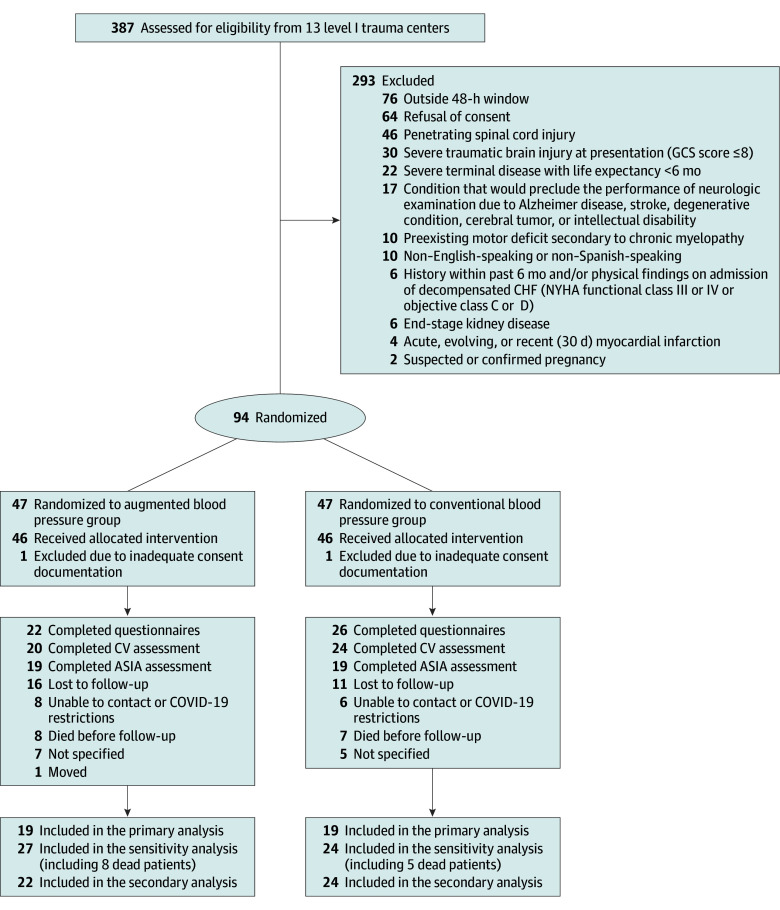
Study Flow Chart Of the 7 patients who died before 6 months in the conventional blood pressure (CBP) group, 2 had missing baseline American Spinal Injury Association (ASIA) scores and were excluded from the sensitivity analysis. CHF indicates congestive heart failure; CV, cardiovascular; GCS, Glasgow Coma Scale; NYHA, New York Heart Association.

A total of 92 patients (mean [SD] age, 53.78 [18.74] years; 76 [83%] male and 16 [17%] female; 12 [13%] Hispanic, 17 [18%] non-Hispanic Black, 56 [61%] non-Hispanic White, and 7 [8%] other) were included (n = 46 per group). From 387 patients who were screened for eligibility, 94 patients met eligibility criteria and were enrolled in the study (Figure 1). Two patients were excluded due to inadequate consent documentation. Due to slow enrollment, the trial was terminated early before reaching the targeted sample size. At baseline, 3 patients had missing sensory and motor scores in the ABP group (2 were intubated before full assessment; complete neurologic examination was unobtainable for 1). At 6 months, 15 patients (16%) had died, 38 (41%) had completed the ASIA assessment, 48 (52%) had completed study questionnaires, and 27 (29%) were lost to follow-up.

Demographic characteristics, comorbidities, concomitant injury, hemodynamic status, and baseline ASIA assessment, including neurologic level of injury, impairment, and motor and sensory scores, were well balanced between the treatment groups (Table 1). Baseline characteristics for the complete case sample were also comparable between groups (eTable 1 in Supplement 2).

**Table 1.** Baseline Characteristics of Patients Randomized Into 2 Blood Pressure Target Groups

Characteristic	No. (%) of patients^a^		
	Overall (N = 92)	ABP group (n = 46)	CBP group (n = 46)
Age, mean (SD), y	53.78 (17.74)	53.57 (17.77)	54.00 (17.89)
BMI, mean (SD)	29.14 (5.78)	29.21 (6.25)	29.08 (5.33)
Sex			
Female	16 (17)	8 (17)	8 (17)
Male	76 (83)	38 (83)	38 (83)
Race and ethnicity			
Hispanic	12 (13)	7 (15)	5 (11)
Non-Hispanic Black	17 (18)	12 (26)	5 (11)
Non-Hispanic White	56 (61)	23 (50)	33 (72)
Other^b^	7 (8)	4 (9)	3 (7)
Smoking history			
Never smoked	43 (47)	20 (43)	23 (50)
Former smoker	16 (17)	8 (17)	8 (17)
Current smoker	20 (22)	10 (22)	10 (22)
Unknown	13 (14)	8 (17)	5 (11)
Smoking pack-years, mean (SD)^c^	19.14 (26.58)	19.23 (33.09)	19.06 (20.70)
Baseline comorbidities before spinal cord injury			
Hypertension	32 (35)	17 (38)	15 (33)
Hypotension	2 (2)	1 (2)	1 (2)
Neuropathy	1 (1)	0	1 (2)
Diabetes	6 (7)	4 (9)	2 (4)
Hyperlipidemia	9 (10)	3 (7)	6 (13)
Stroke	3 (3)	1 (2)	2 (4)
Other cardiovascular disorder	1 (1)	1 (2)	0
Asthma	8 (9)	3 (7)	5 (11)
COPD	3 (3)	2 (4)	1 (2)
Sleep apnea	6 (7)	2 (4)	4 (9)
Other pulmonary disease	4 (4)	3 (7)	1 (2)
Concomitant injury			
Total Injury Severity Score, mean (SD)^d^	23.49 (14.54)	24.95 (13.65)	22.02 (15.41)
Baseline hemodynamic status			
Mean NIBP, mean (SD), mm Hg^e^	87.06 (13.00)	86.08 (11.88)	87.93 (13.99)
Heart rate, mean (SD), /min	71.74 (16.56)	72.04 (13.70)	71.43 (19.15)
Mechanical ventilatory support	22 (24)	12 (26)	10 (22)
Total intravenous fluids since admission, mean (SD), mL	3872.89 (3073.71)	4015.14 (3631.91)	3730.64 (2423.47)
Urine output since admission, mean (SD), mL	2356.45 (1818.06)	2297.35 (1998.69)	2415.54 (1637.77)
Fluid balance since admission, mean (SD), mL^f^	1380.89 (2200.14)	1566.54 (2457.06)	1191.12 (1911.55)
Vasopressors present at baseline	52 (56.52)	28 (60.87)	24 (52.17)
Baseline ASIA assessment			
UEMS, mean (SD)^g^	19.92 (15.85)	17.07 (13.40)	22.98 (17.76)
LEMS, mean (SD)^g^	6.47 (12.83)	6.85 (13.56)	6.07 (12.15)
Total sensory score, mean (SD)^g^	82.74 (49.73)	87.83 (54.36)	77.30 (44.24)
Impairment^f^			
Complete	50 (55)	24 (52)	26 (58)
Incomplete	41 (45)	22 (48)	19 (42)
No. missing	1		1
AIS^h^^,^^f^			
A	50 (55)	24 (52)	26 (58)
B	14 (15)	9 (20)	5 (11)
C	27 (30)	13 (28)	14 (31)
NLI^f^			
C1	1 (1)	0 (0)	1 (2)
C2	7 (8)	6 (13)	1 (2)
C3	6 (7)	0 (0)	6 (13)
C4	28 (31)	15 (33)	13 (29)
C5	24 (26)	15 (33)	9 (20)
C6	7 (8)	5 (11)	2 (4)
C7	1 (1)	1 (2)	0
Below C7	17 (19)	4 (9)	13 (29)

Abbreviations: ABP, augmented blood pressure; AIS, ASIA Impairment Scale; ASIA, American Spinal Injury Association Impairment; BMI, body mass index (calculated as weight in kilograms divided by height in meters squared); CBP, conventional blood pressure; COPD, chronic obstructive pulmonary disease; DVT, deep venous thrombosis, LEMS, lower extremity motor score; MI, myocardial infarction; NIBP, noninvasive blood pressure; NLI, neurologic level of injury; UEMS, upper extremity motor score.

^a^ Unless otherwise indicated.

^b^ Other includes American Indian or Alaska Native, Asian, Native Hawaiian or Other Pacific Islander, unknown, or unspecified or not reported.

^c^ The denominator is the sum number of current and past smokers. Data are missing for 3 patients in the overall group and 3 patients in the ABP group.

^h^ ASIA impairment scale: A, complete; B, sensory incomplete; C, motor incomplete with a muscle grade less than 3; D, motor incomplete with a muscle grade of 3 or higher; E, normal; and U, unknown or not recorded.

^e^ Data are missing for 8 patients in the overall group, 4 in the ABP group, and 3 in the CBP group.

^f^ Data are missing for 7 patients in the overall group, 6 in the ABP group, and 1 in the CBP group.

^g^ Data are missing for 1 patient in the overall group and 1 in the CBP group.

^h^ Data are missing for 3 patients in the overall group and 3 in the CBP group.

**Figure 2.**
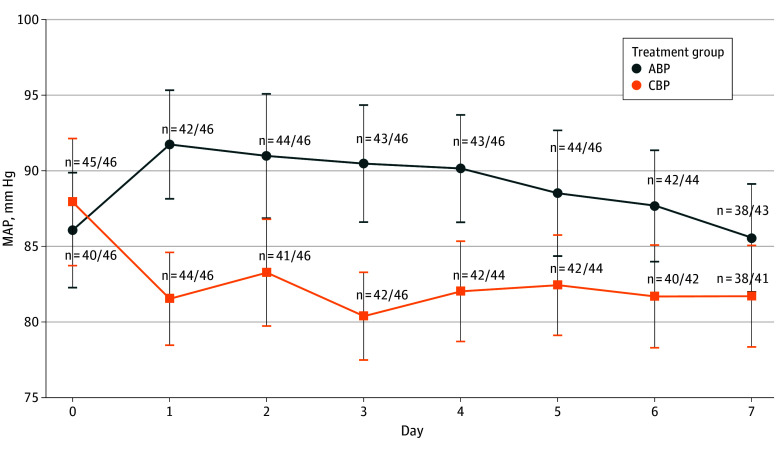
Mean Arterial Pressure (MAP) During the 7-Day Study Period Sample sizes represent the number of patients with MAP monitoring data in the numerator and the total number of patients studied daily in each blood pressure control group. Daily MAP values are the mean of MAP recordings every 4 hours in the intensive care unit or acute care setting from screening (before initiating study protocols) until study day 7 or intensive care unit discharge. MAP values were significantly lower in the conventional blood pressure (CBP) group (>65-70 mm Hg) than the augmented blood pressure (ABP) group (>85-90 mm Hg) during the 7 protocol-specified days (*P* < .001 by repeated-measures regression analysis), noting that the target MAPs were set as lower limit targets that the achieved MAP values were allowed to exceed. The whiskers represent 95% CIs.

Both the ABP and CBP groups maintained MAP targets of greater than 85 to 90 mm Hg and greater than 65 to 70 mm Hg, respectively (Figure 2); however, the average MAP during the first 7 days was greater than 75 mm Hg in general in the CBP group. The mean (SD) duration of intervention was 6.35 (1.46) days in the ABP group and 6.17 (1.77) days in the CBP group (eTable 2 in Supplement 2). Blood pressure targets needed to be temporarily adjusted for 3 patients in the ABP group and for 1 patient in the CBP group, resulting in temporary treatment nonadherence (eTable 2 in Supplement 2).

### Study End Points

**Figure 3.**
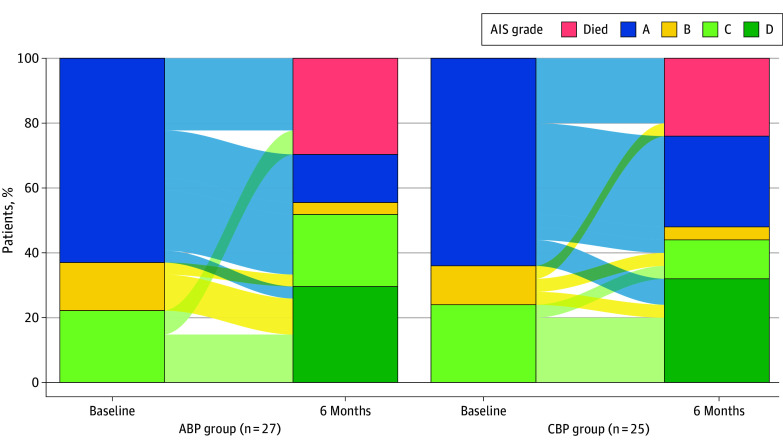
Changes in American Spinal Injury Association (ASIA) Impairment Scale (AIS) Grade at 6 Months ABP indicates augmented blood pressure; CBP, conventional blood pressure.

At 6 months, we did not detect differences between the ABP and CBP groups in the mean (SD) upper extremity motor score (34.95 [3.25] vs 32.95 [3.65]; mean difference, 2.48; 95% CI, −5.93 to 10.90; *P* = .55), lower extremity motor score (18.53 [4.62] vs 19.95 [4.59]; mean difference, −4.56; 95% CI, −16.11 to 7.03; *P* = .43), or total sensory score (108.47 [12.49] vs 130.89 [14.87]; mean difference, −32.00; 95% CI, −65.40 to 1.40; *P* = .06), after adjusting for baseline scores (Table 2). Longitudinal changes in AIS grades are shown in Figure 3. The complete case results were consistent with the sensitivity analyses that imputed scores for 13 patients (5 for CBP and 8 for ABP) who died before follow-up (eTable 3 in Supplement 2). Likewise, there were no noteworthy differences in impairment severity or neurologic level of injury at 6 months (eTable 4 in Supplement 2). There were no differences between groups in pain outcomes, performance in activities of daily living and mobility, satisfaction with the quality of life, or cardiovascular functioning at 6 months (eTable 5 in Supplement 2).

**Table 2.** Complete Case Intention-to-Treat Robust Analysis of Covariance Analysis of ASIA Scores at 6 Months

ASIA scale	Unadjusted group-level scores, mean (SD)				β_1_ (95% CI)^a^	SE	*P* value
	Baseline		6 Months				
	ABP group (n = 19)	CBP (n = 19)	ABP (n = 19)	CBP (n = 19)			
UEMS	19.37 (3.48)	20.31 (3.99)	34.95 (3.25)	32.95 (3.65)	2.48 (−5.93 to 10.90)	4.16	.55
LEMS	7.74 (3.36)	3.79 (2.55)	18.53 (4.62)	19.95 (4.59)	−4.56 (−16.10 to 7.03)	5.71	.43
Total sensory score	102.21 (11.62)	89.26 (9.04)	108.47 (12.49)	130.89 (14.87)	−32.00 (−65.40 to 1.40)	16.45	.06

Abbreviations: ABP, augmented blood pressure; ASIA, American Spinal Injury Association; CBP, conventional blood pressure; LEMS, lower extremity motor score; UEMS, upper extremity motor score.

^a^ Treatment parameter estimate (reference group is the CBP Group) at 6 months, adjusted for the baseline score.

### Safety Outcomes

Four cases were considered nonadherent due to temporary treatment discontinuation (eTable 2 in Supplement 2). Reasons for nonadherence were inability to maintain the study MAP goal (n = 1), subdural hemorrhage (n = 1), and surgery (n = 1) in the ABP group and ventricular tachycardia attributed to high pressor requirements (n = 1) in the CBP group. Patients in the ABP group had higher mean (SD) SOFA scores than the CBP group on day 3 (1.65 [1.79] vs 0.80 [1.10]; mean difference, 0.85; 95% Cl, 0.23-1.47; *P* = .008) and day 6 (1.55 [1.82] vs 0.80 [1.35]; mean difference, 0.74; 95% CI, 0.05-1.44; *P* = .04) (eTable 6 in Supplement 2). The ABP group experienced higher incidence of respiratory complications during hospitalization than the CBP group (36 [78%] vs 18 [39%]; risk difference, 40%; 95% CI, 22%-58%; *P* < .001), specifically pneumonia (20 [45%] vs 11 [25%]; risk difference, 20%; 95% CI, 1%-38%; *P* = .04) and pulmonary edema (10 [22%] vs 3 [7%]; risk difference, 15%; 95% CI, 1%-29%; *P* = .04). The ABP group had longer mean (SD) duration of mechanical ventilatory support than the CBP group (9.44 [15.27] vs 3.78 [8.42] days; mean difference, 5.67 days; 95% CI, 0.48-10.85 days; *P* = .03). No differences were observed in the length of hospital or ICU stays (eTable 5 in Supplement 2). The most frequently reported serious adverse effects at 6 months were death (8 [17%] in the ABP group and 7 [15%] in the CBP group; risk difference, 2%; 95% CI, −13% to 17%; *P* = .82), respiratory complications (8 [17%] in the ABP group and 5 [11%] in the CBP group; risk difference, 6%; 95% CI, −8% to 21%; *P* = .37), and cardiac arrest (3 [7%] in the ABP group and 5 [11%] in the CBP group; risk difference, −4%; 95% CI, −16% to 7%; *P* = .46).

## Discussion

In this multicenter randomized clinical trial of blood pressure augmentation targeting a MAP goal of greater than 85 to 90 mm Hg vs a MAP goal of greater than 65 to 70 mm Hg in patients with acute SCI, we did not detect differences in sensory or motor scores at 6 months. Similarly, we did not detect differences in pain outcomes, performance in activities of daily living, mobility, satisfaction with quality of life, cardiovascular function, death, or serious adverse events at 6 months. Due to early termination and losses to follow-up, the study was underpowered, with the potential for type II error. Nonetheless, patients in the ABP group experienced more ICU treatment-related complications, including a higher incidence of respiratory complications, longer duration of mechanical ventilation, and higher organ dysfunction scores, compared with the CBP group.

Previous observational studies^21,22,23,24,25,26,27,28,29,30,31,32^ reported improved neurologic recovery with higher MAP levels (>85 mm Hg) after SCI. A study^29^ of 92 patients with SCI identified a linear relationship between MAP levels and neurologic improvement at 6 months, and another analysis^26^ of 62 patients with SCI found neurologic improvement at discharge with a MAP of 85 mm Hg or higher compared with a MAP less than 85 mm Hg (25.6% vs 13.5%). Moreover, a retrospective study^25^ of 61 patients with SCI reported that failing to maintain a MAP of 85 mm Hg or higher for at least 2 consecutive hours was associated with higher likelihood of no AIS improvement at 27 days (odds ratio, 11.1; 95% CI, 1.6-75.6) compared with patients maintaining a MAP of 85 mm Hg or higher. Likewise, a retrospective analysis^30^ of 25 patients with SCI revealed that patients who maintained an intraoperative MAP between 70 and 94 mm Hg were more likely to have motor score improvements after discharge, whereas higher or lower MAP ranges were not beneficial.

The MAP range of 75 to 85 mm Hg observed in the CBP group suggests that autoregulation remained sufficiently intact to maintain spinal perfusion pressure in several patients. It is possible that better outcomes reported in previous observational studies^26,27,28,29,30,31,32^ among patients maintaining higher blood pressure primarily reflect preservation of autoregulation. Considering prior observations, the lack of effects in the current randomized clinical trial may suggests that avoiding frank hypotension may be the main benefit of blood pressure–focused management, with higher targets less likely to be associated with episodes of hypotension. Whether benefits are derived from avoidance of hypotension vs blood pressure augmentation remains unknown and potentially challenging, yet important, to study.

Consistent with our findings, a recent retrospective study^32^ of 51 adults with SCI comparing 2 MAP targets (>85-90 vs >65-85 mm Hg) reported no notable differences in neurologic recovery during the patient’s ICU stay or during rehabilitation. Taken together, these results lend support to the more recent guidelines recommending less aggressive MAP targets (>75-80 mm Hg) and cautioning against augmenting MAP above 90 to 95 mm Hg.^10,33^

Our findings are particularly relevant given the clinical challenges and implications of maintaining higher MAP levels with the ABP approach. CBP management, which typically prioritizes a MAP range that is easier to achieve and sustain, may reduce health care resource burden and lower the risk of adverse events associated with elevated blood pressure, such as pulmonary edema and infection—complications that were more common in patients with higher MAP targets in our study.

### Limitations

Our study has limitations. First, the sample size was lower than planned, primarily due to a lack of enrollment during the COVID-19 pandemic, which may have underpowered the study. Despite our rigorous efforts for patient retention, approximately one-third of participants were lost to follow-up. Transporting patients to the clinic for the 6-month neurologic examination was challenging, especially because some follow-up visits coincided with pandemic closures, which intensified losses to follow-up. Additionally, missing data for the primary outcome existed at baseline due to inability to perform complete ASIA assessments (eg, intubated patients). Second, because we did not intentionally lower spontaneous blood pressure, the mean MAP values in the CBP group were greater than 80 mm Hg, which could have contributed to the lack of effect between the 2 groups from a perfusion standpoint, adding to the uncertainty regarding differences between spontaneous and vasopressor-induced blood pressure targets. Third, variability between participating sites, including fluid resuscitation protocols, choices of vasopressors, and surgical approaches, may have occurred. Fourth, our findings may have limited generalizability to patients with penetrating SCI, concomitant traumatic brain injury, or pediatric populations. Fifth, we did not assess long-term management strategies, including intensity of rehabilitation, additional surgical interventions, or pharmacotherapy after hospital discharge, which may have modified the effect of our interventions.

## Conclusions

This randomized clinical trial comparing early augmented (>85-90 mm Hg) with conventional (>65-70 mm Hg) MAP targets after SCI did not find differences in sensory or motor neurologic function at 6 months, although the study was likely underpowered. Furthermore, there were no differences in pain outcomes, performance in activities of daily living and mobility, satisfaction with the quality of life, cardiovascular functioning, and serious adverse events at 6 months. However, patients in the ABP group had less favorable safety profiles in the ICU, including a higher incidence of respiratory complications, longer mechanical ventilatory support, and worse organ dysfunction. Overall, our null findings for efficacy along with an increased safety signal call into question the practice of MAP augmentation in patients with SCI. Although our findings are limited by the lack of power hindering our ability to make a robust inference on targeted blood pressure goals, they are hypothesis-raising regarding optimal hemodynamic management. Further research from adequately powered studies is needed to corroborate the efficacy and safety of MAP goals in patients with SCI, identify patient groups who may be more tolerant or benefit from MAP augmentation (eg, patients with decompression), determine potential harm mechanisms to guide precision medicine approaches, and ensure the compatibility of treatment protocols with evolving evidence and practical feasibility.

## Notes

Supplement 1. Trial Protocol

Supplement 2. **eTable 1.** Baseline characteristics of 38 patients with acute SCI included in the complete-case intent-to-treat analysis **eTable 2.** Medical and surgical interventions in 92 patients with SCI randomized into two blood pressure groups **eTable 3.** Sensitivity analysis of ASIA scores at 6 months including patients who died before follow-up **eTable 4.** ASIA measures in 92 patients with acute SCI randomized into two blood pressure target groups **eTable 5.** Secondary outcomes in 92 patients with SCI randomized into two blood pressure groups **eTable 6.** Safety outcomes in 92 patients with SCI randomized into two blood pressure groups

Supplement 3.
Data Sharing Statement
