# Supplementary material for: Early Blood Pressure Targets in Acute Spinal Cord Injury: A Randomized Clinical Trial
Source: JAMA Netw Open. 2025 Sep 18;8(9):e2525364. doi: 10.1001/jamanetworkopen.2025.25364 (PMC12447234; doi:10.1001/jamanetworkopen.2025.25364)
Supplement: Supplement 1. — Trial Protocol [file jamanetwopen-e2525364-s001.pdf]

Supplemental Online Content

Sajdeya R, Yanez ND, Kampp M, et al. Early blood pressure targets in acute spinal cord injury: a randomized clinical trial. *JAMA Netw Open*. 2025;8(9):e2525364. doi:10.1001/jamanetworkopen.2025.25364

eTable 1. Baseline characteristics of 38 patients with acute SCI included in the complete-case intent-to-treat analysis

eTable 2. Medical and surgical interventions in 92 patients with SCI randomized into two blood pressure groups

eTable 3. Sensitivity analysis of ASIA scores at 6 months including patients who died before follow-up

eTable 4. ASIA measures in 92 patients with acute SCI randomized into two blood pressure target groups

eTable 5. Secondary outcomes in 92 patients with SCI randomized into two blood pressure groups

eTable 6. Safety outcomes in 92 patients with SCI randomized into two blood pressure groups

This supplementary material has been provided by the authors to give readers additional information about their work.

© 2025 Sajdeya R et al. *JAMA Network Open*.

eTable 1. Baseline characteristics of 38 patients with acute SCI included in the complete-case intent-to-treat analysis.

Variable	Overall N=38	ABP Group N=19	CBP Group N=19	*P*-Value
**Demographic Characteristics**				
Age in years, mean (SD)	48.34 (15.74)	45.37 (15.00)	51.32 (16.30)	.25
Sex, N (%)				>.99
Female	7 (18)	4 (21)	3 (16)	
Male	31 (82)	15 (79)	16 (84)	
Race/ethnicity, N (%)				.42
Hispanic	7 (18)	4 (21)	3 (16)	
Non-Hispanic Black	8 (21)	6 (32)	2 (11)	
Non-Hispanic White	20 (53)	8 (42)	12 (63)	
Other [a]	3 (8)	1 (5)	2 (11)	
BMI (kg/m^2), mean (SD)	29.80 (6.14)	29.84 (6.87)	29.76 (5.51)	.97
Smoking history, N (%)				.83
Current smoked	9 (24)	6 (32)	3 (16)	
Former smoker	9 (24)	4 (21)	5 (26)	
Never smoker	18 (47)	8 (42)	10 (53)	
Unknown	2 (5)	1 (5)	1 (5)	
Smoking pack-years, mean (SD) [b]	21.07 (30.93)	24.80 (38.30)	16.88 (21.72)	.61
**Baseline Comorbidities before Spinal Cord Injury**				
Hypertension, N (%)	11 (29)	5 (26)	6 (32)	.80
Diabetes, N (%)	2 (5)	1 (5)	1 (5)	>.99
Hyperlipidemia, N (%)	4 (11)	1 (5)	3 (16)	.60
Asthma, N (%)	6 (16)	2 (11)	4 (21)	.66
COPD, N (%)	1 (3)	1 (5)	0 (0)	.49
Sleep Apnea, N (%)	2 (5)	1 (5)	1 (5)	>.99
Other pulmonary disorder, N (%)	1 (3)	0 (0)	1 (5)	>.99
**Concomitant Injury**				
ISS, mean (SD)	24.86 (16.69) (N missing=1)	28.94 (16.78) (N missing=1)	21.00 (16.09)	.15
**Baseline Hemodynamic Status**				
Mean NIBP, mean (SD)	84.97 (13.28)	82.16 (12.21)	87.79 (14.03)	.195
Heart rate (beat/minute), mean (SD)	70.00 (15.97)	74.21 (12.34)	65.79 (18.29)	.105
Vasopressors at baseline, N (%)	21 (55)	12 (63)	9 (47)	.33
Total IV fluids (mL) since admission, mean (SD)	3877.99 (3213.02)	4040.24 (3635.87)	3715.74 (2818.76)	.760
Urine output (mL) since admission, mean (SD)	2233.07 (1743.39)	2306.58 (1954.82)	2159.56 (1554.02)	.799
Fluid balance (mL) since admission, mean (SD)	1543.64 (2395.72)	1622.77 (2380.27)	1464.52 (2473.64)	.842
Endotracheal intubation, N (%)	6 (16)	4 (21)	2 (11)	.66
Mechanical ventilation, N (%)	7 (18)	5 (26)	2 (11)	.41
**Surgical Intervention**				
Non-surgical bed rest and external immobilization, N (%)				.12
Both enforced bed rest and external immobilizing device	15 (39)	11 (58)	4 (21)	
Enforced bed rest	4 (11)	2 (11)	2 (11)	
External immobilizing device	6 (16)	1 (5)	5 (26)	

© 2025 Sajdeya R et al. *JAMA Network Open.*

Variable	Overall N=38	ABP Group N=19	CBP Group N=19	*P*-Value
None	7 (18)	2 (11)	5 (26)	
Unknown	6 (16)	3 (16)	3 (16)	
Surgical procedure – approach, N (%) [a]				**.01**
Anterior open surgical procedure	10 (26)	2 (11)	8 (42)	
Both anterior and posterior open surgical procedure	5 (13)	5 (26)	0 (0)	
Multi-stage procedure (more than one anesthesia event)	1 (3)	0 (0)	1 (5)	
Posterior open surgical procedure	21 (55)	12 (63)	9 (47)	
N/A	1 (3)	0 (0)	1 (5)	
Surgical procedure - open reduction, N (%)				.61
Yes	24 (63)	13 (68)	11 (58)	
No	12 (32)	5 (26)	7 (37)	
Unknown	1 (3)	1 (5)	0 (0)	
N/A	1 (3)	0 (0)	1 (5)	
Surgical procedure - direct decompression of neural elements, N (%)				.66
Yes	30 (79)	15 (79)	15 (79)	
No	5 (13)	2 (11)	3 (16)	
Unknown	2 (5)	2 (11)	0 (0)	
N/A	1 (2)	0 (0)	1 (5)	
Single or multiple spinal column level procedure, N (%)				>.99
Multiple levels	28 (74)	14 (74)	14 (74)	
Single level	9 (24)	5 (26)	4 (21)	
N/A	1 (3)	0 (0)	1 (5)	
**Baseline ASIA Assessment**				
LTR, mean (SD)	25.84 (12.84)	26.42 (13.25)	25.26 (12.75)	.79
LTL, mean (SD)	25.29 (12.18)	27.16 (13.86)	23.42 (10.28)	.35
Total LT, mean (SD)	51.08 (24.48)	53.47 (27.18)	48.68 (21.93)	.55
PPR, mean (SD)	21.95 (11.13)	23.26 (11.88)	20.63 (10.48)	.47
PPL, mean (SD)	22.71 (12.35)	25.47 (13.93)	19.95 (10.18)	.17
Total PP, mean (SD)	44.66 (22.84)	48.74 (25.25)	40.58 (20.00)	.28
Total Sensory Score, mean (SD)	95.74 (45.25)	102.21 (50.66)	89.26 (39.42)	.39
MSR, mean (SD)	12.21 (10.13)	12.11 (9.34)	12.32 (11.13)	.95
MSL, mean (SD)	13.39 (10.11)	15.00 (9.99)	11.79 (10.25)	.34
UEMS, mean (SD)	19.84 (16.12)	19.37 (15.19)	20.32 (17.41)	.86
LEMS, mean (SD)	5.76 (12.94)	7.74 (14.66)	3.79 (11.00)	.32
NLI at baseline, N (%)				.26
C1	1 (3)	0 (0)	1 (5)	
C2	3 (8)	2 (11)	1 (5)	
C3	2 (5)	0 (0)	2 (11)	
C4	11 (29)	4 (21)	7 (37)	
C5	9 (24)	7 (37)	2 (11)	
C6	5 (13)	3 (16)	2 (11)	
C7	1 (3)	1 (5)	0 (0)	
Below C7	6 (16)	2 (11)	4 (21)	
AIS at baseline, N (%) [c]				.61

© 2025 Sajdeya R et al. *JAMA Network Open*.

Variable	Overall N=38	ABP Group N=19	CBP Group N=19	*P*-Value
A	22 (58)	11 (58)	11 (58)	
B	6 (16)	4 (21)	2 (11)	
C	10 (26)	4 (21)	6 (32)	
Impairment at baseline, N (%)				>.99
Complete	22 (58)	11 (58)	11 (58)	
Incomplete	16 (42)	8 (42)	8 (42)	
**6-month ASIA Assessment**				
LTR, mean (SD)	31.05 (14.64)	28.11 (12.04)	34.00 (16.64)	.22
LTL, mean (SD)	30.92 (15.23)	27.95 (13.81)	33.89 (16.35)	.23
Total LT, mean (SD)	61.95 (29.66)	56.00 (25.53)	67.89 (32.89)	.22
PPR, mean (SD)	29.05 (16.00)	26.32 (14.23)	31.79 (17.55)	.30
PPL, mean (SD)	28.66 (16.37)	26.11 (16.40)	31.21 (16.37)	.34
Total PP, mean (SD)	57.71 (31.97)	52.42 (30.19)	63.00 (33.63)	.31
Total Sensory Score, mean (SD)	119.68 (60.12)	108.47 (54.45)	130.89 (64.80)	.26
MSR, mean (SD)	25.92 (14.12)	25.79 (13.88)	26.05 (14.74)	.95
MSL, mean (SD)	27.26 (15.05)	27.68 (14.63)	26.84 (15.85)	.87
UEMS, mean (SD)	33.95 (14.90)	34.95 (14.16)	32.95 (15.93)	.69
LEMS, mean (SD)	19.24 (19.81)	18.53 (20.15)	19.95 (19.99)	.83
NLI, N (%)				.92
C1	3 (8)	2 (11)	1 (5)	
C2	6 (16)	2 (11)	4 (21)	
C3	2 (5)	1 (5)	1 (5)	
C4	7 (18)	4 (21)	3 (16)	
C5	10 (26)	4 (21)	6 (32)	
C7	4 (11)	2 (11)	2 (11)	
Below C7	6 (16)	4 (21)	2 (11)	
AIS, N (%) [c]				.69
A	11 (29)	4 (21)	7 (37)	
B	2 (5)	1 (5)	1 (5)	
C	9 (24)	6 (32)	3 (16)	
D	16 (42)	8 (42)	8 (42)	
Impairment, N (%)				.28
Complete	11 (29)	4 (21)	7 (364)	
Incomplete	27 (71)	15 (78)	12 (63)	

Note: Percent values are column percentages. *P* value from two-sample t-tests for continuous variables and Chi-square or Fisher Exact test for categorical variables as appropriate. [a] Other included American Indian or Alaska Native; Asian; Native Hawaiian or Other Pacific Islander; unknown; unspecified/not Reported. [b] The denominator is the sum number of current and past smokers. [c] AISA impairment scale: A, Complete; B, Sensory incomplete; C, Motor incomplete with a muscle grade <3; D, Motor incomplete with a muscle grade ≥3; E, Normal; U, Unknown/Not Recorded. [d]

Abbreviations: ABP, augmented blood pressure; AIS, ASIA Impairment Scale; ASIA, American Spinal Injury Association Impairment; BMI, body mass index; CBP, conventional blood pressure; COPD, chronic obstructive pulmonary disease; DVT, deep venous thrombosis; IV, intravenous; LEMS, Lower extremity motor score; LT, light touch; LTL, light touch left; LTR, light touch right; MI, myocardial infarction; MSL, motor score left; MSR, motor score right; N, patient count; N/A, not applicable; NIBP, non-invasive blood pressure; NLI, neurological level of injury; PP, pinprick; PPL, pinprick left; PPR, pinprick right; SCI, spinal cord injury; SD, standard deviation; UEMS, Upper extremity motor score.

© 2025 Sajdeya R et al. *JAMA Network Open*.

eTable 2. Medical and surgical interventions in 92 patients with SCI randomized into two blood pressure groups.

Variable	Overall N=92	ABP Group N=46	CBP Group N=46	*P*-Value
Duration of intervention in days, mean (SD) [a]	6.26 (1.62)	6.35 (1.46)	6.17 (1.77)	0.61
Completed seven days of the intervention, N (%)	72 (78.26%)	36 (78.26%)	36 (78.26%)	>.99
Received vasopressors, N (%)	74 (83) (N missing=3)	37 (84) (N missing=2)	37 (82 (N missing=1)	.81
Number of vasopressors received, median (IQR)	1.36 (0.93) (N missing=3)	1.39 (0.89) (N missing=2)	1.33 (0.98) (N missing=1)	.79
Vasopressors received				
Norepinephrine, N (%)	62 (67)	33 (72)	29 (63)	.37
Phenylephrine, N (%)	33 (36)	14 (30)	19 (41)	.28
Vasopressin, N (%)	16 (17)	11 (24)	5 (11)	.10
Dopamine, N (%)	4 (4)	1 (2)	3 (7)	.62
Other Vasopressor, N (%)	6 (7)	2 (4)	4 (9)	.68
Midodrine, N (%)	23 (25) (N missing=3)	14 (30) (N missing=2)	9 (20) (N missing=1)	.23
Received Anti-hypertensive support medication, N (%)	15 (17)	7 (16)	8 (18)	.81
Non-compliant patients, N (%) [b]	4 (4)	3 (7)	1 (2)	.62
New BP target in non-compliant patients, N (%)				
60-65 mmHg	2 (50)	2 (67)	0 (0)	
70 mmHg	1 (25)	1 (33)	0 (0)	
>85 mmHg	1 (25)	0 (0)	1 (100)	
Non-surgical bed rest and external immobilization, N (%)				.31
Both enforced bed rest and external immobilizing device	24 (27)	16 (36)	8 (18)	
Enforced bed rest	12 (13)	5 (11)	7 (16)	
External immobilizing device	14 (16)	6 (14)	8 (18)	
None	17 (19)	6 (14)	11 (24)	
Unknown	22 (25) (N Missing=3)	11 (25) (N missing=2)	11 (24) (N missing=1)	
Surgical procedure – approach, N (%) [c]				.02
Anterior open surgical procedure	22 (26)	8 (19)	14 (33)	
Both anterior and posterior open surgical procedure	7 (8)	7 (17)	0 (0)	
Multi-stage procedure (more than one anesthesia event)	2 (2)	1 (2)	1 (2)	
Posterior open surgical procedure	52 (62)	25 (60)	27 (64)	
Unknown	1 (1)	1 (2)	0 (0)	
Surgical procedure - open reduction, N (%) [c]				.59
Yes	48 (57)	22 (52)	26 (62)	
No	30 (36)	16 (38)	14 (33)	
Unknown	6 (7)	4 (10)	2 (5)	
Surgical procedure - direct decompression of neural elements, N (%) [c]				.05
Yes	69 (82)	37 (88)	32 (76)	
No	11 (13)	2 (5)	9 (21)	
Unknown	4 (5)	3 (7)	1 (2)	
Single or multiple spinal column level procedure, N (%)				.94
Multiple levels	60 (67)	30 (68)	30 (67)	

© 2025 Sajdeya R et al. *JAMA Network Open*.

Variable	Overall N=92	ABP Group N=46	CBP Group N=46	*P*-Value
Single level	22 (25)	10 (23)	12 (27)	
N/A	6 (7)	3 (7)	3 (7)	
Unknown	1 (1)	1 (2)	0 (0)	
	(N missing=3)	(N missing=2)	(N missing=1)	

Note: Percent values are column percentages. *P* value from two-sample t-tests for continuous variables and Chi-square or Fisher Exact test for categorical variables as appropriate. [a] The duration of intervention was calculated as the number of days from the beginning of treatment randomization/assignment to the end of the seventh day or termination of intervention, whichever came first, where a day was considered 24 hours from the beginning of the intervention. [b] Reasons for noncompliance in the ABP Group were the Inability to maintain the study MAP goal (N=1), subdural hemorrhage (N=1), and surgery (N=1), and in the CBP Group ventricular tachycardia attributed to higher pressor requirements (N=1). [c] N in the denominator=84; i.e., number of patients who had the procedure.
Abbreviations: ABP, augmented blood pressure; CBP, conventional blood pressure; N, patient count; N/A, not applicable; BP, blood pressure; SCI, spinal cord injury.

© 2025 Sajdeya R et al. *JAMA Network Open*.

eTable 3. Sensitivity analysis of ASIA scores at six months including patients who died before follow-up.

ASIA Score, mean (SD)	Baseline [a]		6 Months [a]		β_1 (95% CI) [b]	SE	*P*-value
	ABP Group (N=27)	CBP Group (N=24)	ABP Group (N=27)	CBP Group (N=24)			
UEMS	17.48 (2.65)	20.33 (3.52)	34.0 (3.18)	32.8 (3.62)	2.64 (-6.90, 12.2)	4.62	0.57
LEMS	6.19 (2.50)	3.58 (2.06)	17.3 (4.44)	19.5 (4.53)	-4.26 (-17.5, 8.93)	6.38	0.51
Total Sensory Score	84.96 (9.99)	79.88 (8.95)	95.82 (13.3)	123.4 (14.7)	-31.2 (-69.2, 6.89)	18.4	0.10

Note: Parameter estimates from a linear regression model with robust standard error estimates for test statistics and confidence limits. [a] Unadjusted group-level mean (SE) for baseline and follow-up scores. [b] Treatment parameter estimate (reference: CBP Group).
Abbreviations: ABP, augmented blood pressure; ASIA, American Spinal Injury Association; CBP, conventional blood pressure groups; CI, confidence interval; LEMS, lower extremity motor score; LT, light touch; PP, pinprick; SCI, spinal cord injury; SE, standard error; UEMS, upper extremity motor score.

© 2025 Sajdeya R et al. *JAMA Network Open*.

eTable 4. ASIA measures in 92 patients with acute SCI randomized into two blood pressure target groups.

Variable	Overall N=92	ABP Group N=46	CBP Group N=46	*P*-Value
**Baseline ASIA Assessment**				
LTR, mean (SD)	22.78 (14.38) (N missing=3)	23.76 (15.47)	21.72 (13.22) (N missing=3)	.51
LTL, mean (SD)	22.25 (14.24) (N missing=3)	23.96 (15.77)	20.42 (12.30) (N missing=3)	.24
Total LT, mean (SD)	45.00 (28.31) (N missing=3)	47.67 (31.17)	42.14 (24.94) (N missing=3)	.36
PPR, mean (SD)	18.69 (11.97) (N missing=3)	19.43 (12.81)	17.88 (11.09) (N missing=3)	.54
PPL, mean (SD)	19.06 (13.23) (N missing=3)	20.72 (14.44)	17.28 (11.70) (N missing=3)	.22
Total PP, mean (SD)	37.74 (24.71) (N missing=3)	40.15 (26.82)	35.16 (22.26) (N missing=3)	.34
Total Sensory Score, mean (SD)	82.74 (49.73) (N missing=3)	87.83 (54.36)	77.30 (44.24) (N missing=3)	.32
UEMS, mean (SD)	19.92 (15.85) (N missing=3)	17.07 (13.40)	22.98 (17.76) (N missing=3)	.08
LEMS, mean (SD)	6.47 (12.83) (N missing=3)	6.85 (13.56)	6.07 (12.15) (N missing=3)	.78
MSR, mean (SD)	12.89 (10.37) (N missing=3)	10.83 (9.02)	15.09 (11.33) (N missing=3)	.05
MSL, mean (SD)	13.51 (9.76) (N missing=3)	13.09 (9.77)	13.95 (9.84) (N missing=3)	.68
Impairment, N (%)				.53
Complete	50 (55)	24 (52)	26 (58)	
Incomplete	41 (45) (N missing=1)	22 (48)	19 (42) (N missing=1)	
AIS, N (%) [b]				.54
A	50 (55)	24 (52)	26 (58)	
B	14 (15)	9 (20)	5 (11)	
C	27 (30) (N missing=1)	13 (28)	14 (31) (N missing=1)	
NLI, N (%)				**.003**
C1	1 (1)	0 (0)	1 (2)	
C2	7 (8)	6 (13)	1 (2)	
C3	6 (7)	0 (0)	6 (13)	
C4	28 (31)	15 (33)	13 (29)	
C5	24 (26)	15 (33)	9 (20)	
C6	7 (8)	5 (11)	2 (4)	
C7	1 (1)	1 (2)	0 (0)	
Below C7	17 (19) (N missing=1)	4 (9)	13 (29) (N missing=1)	
**6 Months ASIA Assessment**				
LTR, mean (SD)	31.05 (14.64) (N missing=54)	28.11 (12.04) (N missing=27)	34.00 (16.64) (N missing=27)	.22
LTL, mean (SD)	30.92 (15.23) (N missing=54)	27.95 (13.81) (N missing=27)	33.89 (16.35) (N missing=27)	.23
Total LT, mean (SD)	61.95 (29.66) (N missing=54)	56.00 (25.53) (N missing=27)	67.89 (32.89) (N missing=27)	.22

© 2025 Sajdeya R et al. *JAMA Network Open*.

Variable	Overall N=92	ABP Group N=46	CBP Group N=46	*P*-Value
PPR, Mean (SD)	29.05 (16.00) (N missing=54)	26.32 (14.23) (N missing=27)	31.79 (17.55) (N missing=27)	.30
PPL, Mean (SD)	28.66 (16.37) (N missing=54)	26.11 (16.40) (N missing=27)	31.21 (16.37) (N missing=27)	.34
Total PP, Mean (SD)	57.71 (31.97) (N missing=54)	52.42 (30.19) (N missing=27)	63.00 (33.63) (N missing=27)	.31
Total Sensory Score, Mean (SD)	119.68 (60.12) (N missing=54)	108.47 (54.45) (N missing=27)	130.89 (64.80) (N missing=27)	.26
UEMS, Mean (SD)	33.95 (14.90) (N missing=54)	34.95 (14.16) (N missing=27)	32.95 (15.93) (N missing=27)	.69
LEMS, Mean (SD)	19.24 (19.81) (N missing=54)	18.53 (20.15) (N missing=27)	19.95 (19.99) (N missing=27)	.83
MSR, Mean (SD)	25.92 (14.12) (N missing=54)	25.79 (13.88) (N missing=27)	26.05 (14.74) (N missing=27)	.96
MSL, Mean (SD)	27.26 (15.05) (N missing=54)	27.68 (14.63) (N missing=27)	26.84 (15.85) (N missing=27)	.87
Impairment, N (%)				.28
Complete	11 (29)	4 (21)	7 (37)	
Incomplete	27 (71)	15 (78)	12 (63)	
	(N missing=54)	(N missing=27)	(N missing=27)	
AIS, N (%) [b]				.69
A	11 (29)	4 (21)	7 (37)	
B	2 (5)	1 (5)	1 (5)	
C	9 (24)	6 (32)	3 (16)	
D	16 (42)	8 (42)	8 (42)	
	(N missing=54)	(N missing=27)	(N missing=27)	
NLI, N (%)				.92
C1	3 (8)	2 (11)	1 (5)	
C2	6 (16)	2 (11)	4 (21)	
C3	2 (5)	1 (5)	1 (5)	
C4	7 (18)	4 (21)	3 (16)	
C5	10 (26)	4 (21)	6 (32)	
C7	4 (11)	2 (11)	2 (11)	
Below C7	6 (16)	4 (21)	2 (11)	
	(N missing=54)	(N missing=27)	(N missing=27)	

Note: Percent values are column percentages. P value from two-sample t-tests for continuous variables and Chi-square or Fisher Exact test for categorical variables as appropriate. [a] Spinal cord level; [b] ASIA impairment scale: A, Complete; B, Sensory incomplete; C, Motor incomplete with a muscle grade <3; D, Motor incomplete with a muscle grade ≥3; E, Normal; U, Unknown/Not Recorded

Abbreviations: ABP, augmented blood pressure; AIS, ASIA Impairment Scale; ASIA, American Spinal Injury Association Impairment; CBP, conventional blood pressure; LEMS, Lower extremity motor score; LT, light touch; LTL, light touch left; LTR, light touch right; MSL, motor score left; MSR, motor score right; N, patient count; NLI, neurological level of injury; PP, pinprick; PPL, pinprick left; PPR, pinprick right; SCI, spinal cord injury; UEMS, Upper extremity motor score.

© 2025 Sajdeya R et al. *JAMA Network Open*.

eTable 5. Secondary outcomes in 92 patients with SCI randomized into two blood pressure groups.

Variable	Overall N=92	ABP Group N=46	CBP Group N=46	*P*-Value
**ISCIPBDS**				
Pain the last seven days, N (%)	40 (83) (N missing=44)	18 (82) (N missing=24)	22 (852) (N missing=20)	>.99
Interference of pain with daily activity score, mean (SD)	5.02 (3.40) (N missing=44)	5.21 (3.17) (N missing=24)	4.86 (3.66) (N missing=20)	.75
Interference of pain with mood score, mean (SD)	5.10 (3.47) (N missing=45)	5.05 (3.15) (N missing=24)	5.14 (3.81) (N missing=21)	.94
Interference of pain with sleep score, mean (SD)	4.88 (3.53) (N missing=45)	5.21 (3.22) (N missing=24)	4.57 (3.84) (N missing=21)	.57
Number of different pain problems, N (%)				.59
One	10 (24)	4 (22)	6 (26)	
Two	12 (29)	6 (33)	6 (26)	
Three	13 (32)	7 (39)	6 (26)	
Four	3 (7)	0 (0)	3 (13)	
Five or more	3 (7) (N missing=44)	1 (6) (N missing=24)	2 (9) (N missing=20)	
Pain #1 (N=41) [a]				
ISCIBP total score, mean (SD)	12.12 (7.55) (N missing=15)	9.38 (8.38) (N missing=11)	13.33 (7.06) (N missing=4)	.23
Pain location, N (%) [b]				
Head	2 (5)	2 (11)	0 (0)	.19
Neck and throat	13 (32)	8 (44)	5 (22)	.12
Upper extremity	16 (47)	6 (38)	10 (56)	.29
Chest	2 (5)	1 (6)	1 (4)	>.99
Abdomen and pelvis	2 (5)	1 (6)	1 (4)	>.99
Back	11 (27)	6 (33)	5 (224)	.49
Lower extremity	10 (24)	3 (17)	7 (30)	.47
Type of pain, N (%)				.29
Neuropathic	7 (18)	1 (6)	6 (27)	
Nociceptive	19 (50)	9 (56)	10 (45)	
Unclear	12 (32) (N missing=3)	6 (380) (N missing=2)	6 (27) (N missing=1)	
Pain intensity, mean (SD)	6.20 (2.24)	6.39 (2.23)	6.04 (2.29)	.63
Received pain treatment, N (%)	34 (83)	16 (89)	18 (78)	.44
Pain #2 (N=31) [c]				
ISCIBP total score, mean (SD)	9.62 (6.75) (N missing=10)	10.43 (5.35) (N missing=7)	9.21 (7.51) (N missing=3)	.71
Type of pain, N (%)				.29
Neuropathic	6 (22)	2 (17)	4 (27)	
Nociceptive	16 (59)	7 (58)	9 (60)	
Unclear	5 (19) (N missing=4)	3 (25) (N missing=2)	2 (13) (N missing=2)	
Pain intensity, mean (SD)	5.55 (2.14)	5.21 (2.26)	5.82 (2.07)	.44
Received pain treatment, N (%)	24 (77)	10 (71)	14 (82)	.67
Pain #3 (N=19) [d]				
ISCIBP total score, mean (SD)	10.77 (8.80) (N missing=6)	10.00 (6.68) (N missing=4)	11.11 (9.94) (N missing=2)	.84
Type of pain, N (%)				>.99
Neuropathic	1 (6)	0 (0)	1 (10)	

© 2025 Sajdeya R et al. *JAMA Network Open*.

Variable	Overall N=92	ABP Group N=46	CBP Group N=46	*P*-Value
Nociceptive	8 (50)	3 (50)	5 (50)	
Unclear	7 (44) (N missing=3)	3 (50) (N missing=2)	4 (40) (N missing=1)	
Pain intensity, mean (SD)	6.06 (2.16) (N missing=2)	6.00 (1.73) (N missing =1)	6.10 (2.51) (N missing=1)	.93
Received pain treatment, N (%)	14 (78) (N missing=1)	5 (71) (N missing=1)	9 (82)	>.99
**SCIM III Score, mean (SD) [e]**	27.83 (20.01) (N missing=44)	27.48 (19.43) (N missing=24)	28.11 (20.82) (N missing=20)	.92
**ISCIQOL**				
How satisfied are you with your physical health in the past four weeks? Mean (SD)	6.07 (2.48) (N missing=50)	6.44 (2.25) (N missing=28)	5.79 (2.65) (N missing=22)	.41
Thinking about your own life and personal circumstances, how satisfied are you with your life as a whole in the past four weeks? Mean (SD)	5.98 (2.72) (N missing=51)	6.47 (2.46) (N missing=27)	5.55 (2.92) (N missing=24)	.28
How satisfied are you with your psychological health, emotions, and mood in the past four weeks? Mean (SD)	6.55 (2.91) (N missing=50)	6.94 (2.41) (N missing=29)	6.28 (3.22) (N missing=21)	.48
**Events related to cardiovascular function after spinal cord lesion**				
Cardiac Pacemaker, N (%)	4 (9) (N missing=49)	1 (5) (N missing=27)	3 (13) (N missing=22)	.62
Stroke, N (%)	1 (2) (N missing=49)	1 (5) (N missing=27)	0 (0) (N missing=22)	.44
DVT, N (%)	5 (12) (N missing=49)	4 (21) (N missing=27)	1 (4) (N missing=22)	.15
**Cardiovascular function after spinal cord lesion within the last three months**				
Cardiac conditions, N (%)	1 (2) (N missing=49)	0 (0) (N missing=27)	1 (4) (N missing=22)	>.99
Pulmonary embolism, N (%)	1 (2) (N missing=49)	1 (5) (N missing=27)	0 (0) (N missing=22)	.44
Orthostatic hypotension, N (%)	5 (12) (N missing=49)	2 (11) (N missing=27)	3 (13) (N missing=22)	>.99
Dependent edema, N (%)	3 (7) (N missing=49)	1 (5) (N missing=27)	2 (8) (N missing=22)	>.99
Hypertension, N (%)	2 (5) (N missing=49)	1 (5) (N missing=27)	1 (4) (N missing=22)	>.99
Autonomic dysreflexia, N (%)	6 (14) (N missing=49)	1 (5) (N missing=27)	5 (21) (N missing=22)	.21
DVT, N (%)	1 (2) (N missing=49)	1 (5) (N missing=27)	0 (0) (N missing=22)	.44
Other, N (%)	1 (2) (N missing=49)	1 (5) (N missing=27)	0 (0) (N missing=22)	.44
**Any medication affecting cardiovascular function on the day of examination?**				
Anticholinergic, N (%)	3 (7) (N missing=49)	2 (11) (N missing=27)	1 (4) (N missing=22)	.58

© 2025 Sajdeya R et al. *JAMA Network Open*.

Variable	Overall N=92	ABP Group N=46	CBP Group N=46	*P*-Value
Antihypertensives (beta-blocker, antiarrhythmics, ACE, etc.)	6 (14) (N missing=49)	1 (5) (N missing=27)	5 (21) (N missing=22)	.21
Antihypotensives, N (%)	6 (14) (N missing=49)	2 (11) (N missing=27)	4 (17) (N missing=22)	.68
Cardiac medications (e.g., digitalis, etc.), N (%)	3 (7) (N missing=49)	1 (5) (N missing=27)	2 (8) (N missing=22)	>.99
Other, N (%)	1 (2) (N missing=49)	0 (0) (N missing=27)	1 (4) (N missing=22)	>.99
**Vital Signs**				
Heart rate, mean (SD)	74.79 (13.24) (N Missing=59)	74.93 (14.60) (N missing=31)	74.67 (12.43) (N missing=28)	.96
Systolic BP, mean (SD)	116.45 (18.50) (N missing=59)	115.07 (20.22) (N missing=31)	117.61 (17.45) (N missing=28)	.70
Diastolic BP, mean (SD)	72.52 (12.07) (N missing=59)	72.20 (12.60) (N missing=31)	72.78 (11.96) (N missing=28)	.89
Mean BP, mean (SD)	86.65 (14.75) (N missing=59)	86.42 (15.60) (N missing=31)	86.84 (14.45) (N missing=28)	.94

Note: Percent values are column percentages. *P* value from two-sample t-tests for continuous variables and Chi-square or Fisher Exact test for categorical variables as appropriate. [a] N in the denominator is the number of people who reported having at least 1 pain (N=41). [b] Answers are not mutually exclusive; total percentages do not equal 100%. [c] N in the denominator is the number of people who reported having at least 2 pains (N=31). [d] N in the denominator is the number of people who reported having at least 3 pains (N=19). [e] The SCIM-III is used to assess performance in activities of daily living and mobility in individuals with spinal cord injury. The SCIM is composed of 19 items that assess 2 domains. 1) Self-care (6 items, scores range from 0-20): a) feeding. b) bathing. c) dressing, d) grooming. 2) Respiration and sphincter management (4 items, scores range from 0-40): a) Respiration, b) bladder management, c) bowel management, d) use of toilet. 3) Mobility (9 items, scores range from 0-40: a) Tasks in the room and toilet, b) tasks indoors and outdoors. The total SCIM scores range from 0 to 100, where a score of 0 defines total dependence, and a score of 100 is indicative of complete independence.
Abbreviations: ABP, augmented blood pressure; CBP, conventional blood pressure; DVT, deep venous thrombosis; ISCIPBDS, International Spinal Cord Injury Pain Basic Data Set; ISCIQOL, International Society of Quality of Life; N, patient count; SCI, spinal cord injury; SCIM III; Spinal Cord Independence Measure, Version III; SD, standard deviation.

© 2025 Sajdeya R et al. *JAMA Network Open*.

eTable 6. Safety outcomes in 92 patients with SCI randomized into two blood pressure groups.

Variable	Overall N=92	ABP Group N=46	CBP Group N=46	*P*-Value
**Safety Outcomes During Hospitalization**				
Tracheostomy, N (%)	25 (28) (N missing=2)	14 (31) (N missing=1)	11 (24) (N missing=1)	.48
Utilization of ventilator assistance on discharge, N (%)				.25
No	72 (80)	33 (73)	39 (87)	
Yes	15 (16)	10 (22)	5 (11)	
Unknown	5 (5)	3 (7)	2 (4)	
Respiratory complications, N (%) [a]	54 (59) (N missing=2)	36 (78) (N missing=1)	18 (39) (N missing=1)	**<.001**
ARDS in ICU	13 (14) (N missing=2)	8 (18) (N missing=1)	5 (11) (N missing=1)	.37
Pneumonia	31 (35) (N missing=2)	20 (45) (N missing=1)	11 (25) (N missing=1)	**.04**
Pulmonary edema	13 (14) (N missing=2)	10 (22) (N missing=1)	3 (7) (N missing=1)	**.04**
Administered dexamethasone, N (%)	36 (40)	19 (42)	17 (38)	.67
Vital status on discharge, N (%)				.43
Alive	83 (92)	40 (89)	42 (93)	
Dead	7 (8) (N missing=2)	5 (11) (N missing=1)	3 (7) (N missing=1)	
Discharge disposition, N (%)				.07
Inpatient rehabilitation unit	52 (57)	19 (42)	33 (72)	
Long-term acute care facility	18 (20)	11 (24)	7 (15)	
Nursing home	8 (9)	5 (11)	3 (7)	
Deceased	8 (9)	5 (11)	3 (7)	
Other hospital	3 (3)	3 (7)	0 (0)	
Other/ unclassified/ Unknown	2 (2) (N missing=1)	2 (4) (N missing=1)	0 (0)	
Length of hospital stay in days, mean (SD)	18.62 (14.01) (N missing=3)	18.87 (12.55) (N missing=1)	18.36 (15.51) (N missing=2)	.87
Length of ICU stay in days, mean (SD)	11.85 (10.42) (N missing=1)	12.80 (8.19) (N missing=1)	10.91 (12.23)	.39
Duration of mechanical ventilation in days, mean (SD)	6.61 (12.58) (N missing=2)	9.44 (15.27) (N missing=1)	3.78 (8.42) (N missing=1)	**.03**
Utilization of ventilator assistance on discharge, N (%)	15 (17%) (N missing=5)	10 (23%) (N missing=3)	5 (11%) (N missing=2)	.14
SOFA score, mean (SD) [b]				
Day 1	1.52 (1.63) (N=91/92)	1.80 (1.83) (N=46/46)	1.22 (1.35) (N=45/46)	.09
Day 2	1.42 1.49 (N=92/92)	1.65 (1.69) (N=46/46)	1.20 (1.24) (N=46/46)	.14
Day 3	1.23 (1.54) (N=91/92)	1.65 (1.79) (N=46/46)	0.80 (1.10) (N=45/46)	**.008**
Day 4	1.39 (1.50) (N=89/90)	1.63 (1.58) (N=46/46)	1.14 (1.37) (N=43/44)	.12
Day 5	1.22 (1.44) (N=89/90)	1.30 (1.44) (N=46/46)	1.14 (1.44) (N=43/44)	.59

© 2025 Sajdeya R et al. *JAMA Network Open.*

Variable	Overall N=92	ABP Group N=46	CBP Group N=46	*P*-Value
Day 6	1.19 (1.64) (N=85/88)	1.55 (1.82) (N=44/44)	0.80 (1.35) (N=41/42)	**.04**
Day 7	1.10 (1.64) (N=83/84)	1.40 (1.84) (N=43/43)	0.78 (1.33) (N=40/41)	.08
**Serious Adverse Events at six months**				
Any serious adverse event, N (%) [c]	25 (27)	12 (26)	13 (28)	.82
Fatal adverse events, N (%)	15 (16)	8 (17)	7 (15)	.78
Cardiac arrest, N (%)	8 (9)	3 (7)	5 (11)	.46
Respiratory complications, N (%) [d]	13 (14)	8 (17)	5 (11)	.37
Other serious adverse events, N (%) [e]	10 (11)	5 (11)	5 (11)	>.99

Note: Percent values are column percentages. *P* value from two-sample t-tests for continuous variables and Chi-square or Fisher Exact test for categorical variables as appropriate. [a] A composite of acute respiratory distress syndrome, hypoxemia as indicated by PaO2/FiO2 ratio, and pneumonia. [b] Modified SOFA score was calculated by excluding the “mean arterial pressure or administration of vasoactive agents required” since the study intervention determined MAP goals. Number of patients in the denominator changed per (e.g., patient discharge), and N is displayed as the number of patients with no missing data over the total number of patients per day. [c] Some patients had multiple serious adverse events, some of which were fatal. [d] Serious respiratory complications included pneumonia, respiratory failure, hypoxia, and atelectasis. [e] Other serious adverse events included sepsis/septic shock, stroke, perirectal ulcer with necrosis, brain hemorrhage, urinary tract infection, and C. difficile infection.
Abbreviations: ABP, augmented blood pressure; ARDS, acute respiratory distress syndrome; CBP, conventional blood pressure; ICU, intensive care unit; N, patient count; SCI, spinal cord injury; SOFA, sequential organ failure assessment.

© 2025 Sajdeya R et al. *JAMA Network Open*.
