# Supplementary material for: Early Blood Pressure Targets in Acute Spinal Cord Injury: A Randomized Clinical Trial
Source: JAMA Netw Open. 2025 Sep 18;8(9):e2525364. doi: 10.1001/jamanetworkopen.2025.25364 (PMC12447234; doi:10.1001/jamanetworkopen.2025.25364)
Supplement: Supplement 2. — eTable 1. Baseline characteristics of 38 patients with acute SCI included in the complete-case intent-to-treat analysis eTable 2. Medical and surgical interventions in 92 patients with SCI randomized into two blood pressure groups eTable 3. Sensitivity analysis of ASIA scores at 6 months including patients who died before follow-up eTable 4. ASIA measures in 92 patients with acute SCI randomized into two blood pressure target groups eTable 5. Secondary outcomes in 92 patients with SCI randomized into two blood pressure groups eTable 6. Safety outcomes in 92 patients with SCI randomized into two blood pressure groups [file jamanetwopen-e2525364-s002.pdf]

Randomized Trial of Early Hemodynamic Management of Patients following Acute Spinal Cord Injury (TEMPLE)

Protocol Number: TEMPLE - 004

Grant Number: SC150250

Principal Investigator: Miriam Treggiari, MD, PhD, MPH

Co- Principal Investigator: David Zonies, MD, MPH

Research Monitor: Peter Schulman, MD

Biostatistician: N. David Yanez, PhD

Version Number: 4.1

08 AUGUST 2018

STATEMENT OF COMPLIANCE

The study will be conducted in accordance with the International Conference on Harmonisation guidelines for Good Clinical Practice (ICH E6), the Code of Federal Regulations on the Protection of Human Subjects (45 CFR Part 46), and the Department of Defense Terms of Award. All personnel involved in the conduct of this study have completed human subjects protection training.

SIGNATURE PAGE

The signature below constitutes the approval of this protocol and the attachments, and provides the necessary assurances that this trial will be conducted according to all stipulations of the protocol, including all statements regarding confidentiality, and according to local legal and regulatory requirements and applicable US federal regulations and ICH guidelines.

Principal Investigator or Clinical Site Investigator:

Signed: ______________________________ Date: ______________

Name: ______________________________

Title: ______________________________

Contents

STATEMENT OF COMPLIANCE I
SIGNATURE PAGE II
LIST OF ABBREVIATIONS VI
PROTOCOL SUMMARY VIII
KEY ROLES AND CONTACT INFORMATION 10
1 ETHICS 11
1.1 Ethical Standard 11
1.2 Institutional Review Boards 11
1.3 Subject Information and Informed Consent 11
1.4 Patient Confidentiality 11
1.5 Protocol Compliance 12
2 INVESTIGATORS AND TRIAL ADMINISTRATIVE STRUCTURE 13
2.1 Investigators and Trial Site Personnel 13
2.1.1 Investigators 13
2.1.2 Trial Site Personnel Assigned Trial-Related Duties 13
2.2 Data Monitoring Committee 13
3 INTRODUCTION 14
3.1 Background Information 14
3.2 Specific Aims / Study Design 15
3.3 Potential Risks and Benefits 15
3.3.1 Potential Risks 15
3.3.2 Frequency of TPM Associated Risks 16
3.3.3 Mitigation of Potential Risks 16
3.3.4 Potential Benefits 17
4 OBJECTIVES 18
4.1 Study Objectives 18
4.2 Study Outcome Measures 18
4.2.1 Primary Specific Aim 18
4.2.2 Secondary Specific Aim for Efficacy 18
4.2.3 Specific Aims for Safety 18
5 STUDY DESIGN 19
5.1 Setting 19
STUDY ENROLLMENT AND WITHDRAWAL 20
5.2 Subject Enrollment Procedure 20
5.3 Inclusion/ Exclusion Criteria 20
5.3.1 Inclusion Criteria 20
5.3.2 Exclusion Criteria 21
5.3.3 Inclusion of Women and Minorities 21
5.3.4 Exclusion of Vulnerable Populations 22
5.4 Strategies for Recruitment 22
5.5 Informed Consent Process 22
5.6 Point of Enrollment 23

5.6.1 Subject Trial Cards 23
5.6.2 Subject Retention 23
5.7 Treatment Assignment Procedures 24
5.7.1 Randomization Procedures 24
5.7.2 Blinding 24
5.8 Subject Discontinuation 25
5.8.1 Investigator Initiated Withdrawal 25
5.8.2 Subject Initiated Withdrawal 25
5.8.3 Procedures for Handling Discontinued Subjects 25
5.9 Premature Termination or Suspension of Study 25
6 STUDY INTERVENTION 27
6.1 Study Treatment Protocols 27
6.1.1 Targeted blood Pressure Management 28
6.1.2 Volume Expansion to Achieve Euvolemia 28
6.2 Assessment of Clinicians' Compliance with Study Procedural Intervention 28
7 STUDY SCHEDULE AND EVALUATIONS 29
7.1 Pre-randomization Assessment 29
7.2 ICU Visit Assessment 29
7.2.1 Concomitant Treatments and Supportive Care 29
7.2.2 Documentation of post-72 hour ASIA Score 30
7.2.3 Hospital Discharge 30
7.2.4 Adverse Event Monitoring 31
7.3 Six Month Follow-Up Assessment 31
7.3.1 Patient Tracking at Follow-Up 31
8 ASSESSMENT OF SAFETY 32
8.1 Specification of Safety Parameters 32
8.1.1 Unanticipated Problems 32
8.1.2 Protocol Deviation 32
8.1.3 Adverse Events 33
8.1.4 Serious Adverse Events 33
8.2 Characteristics of an Adverse Event 33
8.2.1 Relationship to Study Intervention 33
8.2.2 Expectedness of SAEs 34
8.2.3 Severity of Event 34
8.2.4 Outcome of Event 34
8.3 Time Period and Frequency for Event Assessment and Follow-Up 34
8.4 Reporting Procedures 35
8.4.1 Unanticipated Problem Reporting 35
8.4.2 Serious Adverse Event Reporting 35
8.5 Stopping Rules 36
9 STUDY OVERSIGHT 37
9.1 Quality System 37
9.2 Promoting and Monitoring Adherence to the Research Protocol 37

9.3 Monitoring of Study Conduct 37
9.4 Study Monitoring and Site Visits 38
9.5 Adverse Event Monitoring 38
9.6 Research Monitor 39
9.7 Data Safety and Monitoring Board 39
9.8 Steering Committee 40
9.9 Audits 40
9.10 Inspections 40
10 STATISTICAL CONSIDERATIONS 41
10.1 Assessment of Study Variables 41
10.1.1 Primary Endpoint 41
10.1.2 Secondary Endpoints for Efficacy 41
10.1.3 Secondary Endpoints for Safety 42
10.2 Sample Size Estimation and Statistical Power 44
10.3 Statistical Analysis Plan 45
11 DATA HANDLING AND RECORD KEEPING 47
11.1 Data Management Responsibilities 47
11.2 Source Data 47
11.3 Case Report Forms 47
11.4 Identifiers 48
11.5 Confidentiality 48
11.6 Data Entry Completion 49
11.7 Study Records Retention 49
11.8 Data and Resources Sharing Plan 50
11.9 Data Quality Assurance 50
12 PUBLICATION/DATA SHARING POLICY 51
13 LITERATURE REFERENCES 52
APPENDIX A: SCHEDULE OF EVENTS 53
APPENDIX B: INTERVENTION ALGORITHMS 55

LIST OF ABBREVIATIONS

ABP	Augmented Blood Pressure
AE	Adverse Event/Adverse Experience
AIS	ASIA Impairment Scale
ASIA	American Spinal Injury Association
CBP	Conventional Blood Pressure
CFR	Code of Federal Regulations
CRF	Case Report Form
CT	Computerized Axial Tomography
CVP	Central Venous Pressures
DHHS	Department of Health and Human Services
DMU	Data Management Unit
DSMB	Data and Safety Monitoring Board
eCRF	Electronic Case Report Form
EKG	Electrocardiogram
EMR	Electronic Medical Record
FDA	Food and Drug Administration
GCP	Good Clinical Practice
HR	Heart Rate
HIPAA	Health Insurance Portability and Accountability Act
ICH	International Conference on Harmonisation
ICU	Intensive Care Unit
IDE	Investigational Device Exemption
IND	Investigational New Drug Application
IRB	Institutional Review Board
ISCIBPDS	International Spinal Cord Injury Basic Pain Data Set
ISCIQOL	International Spinal Cord Injury Quality of Life Basic Data Set
LAR	Legally Authorized Representative
MAP	Mean Arterial Pressure
MOO	Manual of Operations
MRI	Magnetic Resonance Imaging
NIDCR	National Institute of Dental and Craniofacial Research, NIH, DHHS
NIH	National Institutes of Health
OHSU	Oregon Health and Science University

PI	Principal Investigator
PP	Pin Prick
SAE	Serious Adverse Event/Serious Adverse Experience
SAH	Subarachnoid Hemorrhage
SCI	Spinal Cord Injury
SCIM III	Spinal Cord Independence Measure III
SOFA	Sequential Multiple Organ Failure Score
SOP	Standard Operating Procedure
TPM	Targeted blood Pressure Management

PROTOCOL SUMMARY

**Title:**	Randomized Trial of Early Hemodynamic Management of Patients following Acute Spinal Cord Injury (TEMPLE)
**Study Design**	This is a multicenter, randomized, controlled study. Subjects will be randomized 1:1 to one of two treatment arms, monitored for up to 7 days, and assessed at a 6 month follow up visit.
**Objectives:**	Primary: To determine the differential effects of two levels of MAP on long-term motor and sensory outcomes after acute SCI.
	Secondary: To determine the differential efficacies of two levels of MAP based on long-term pain and functional independence measures.
	Safety: To determine the differential safety profiles of maintaining two different levels of MAP for up to 7 days.
**Population:**	Adult patients of either gender with a diagnosis of acute traumatic spinal cord injury involving neurological levels.
**Sample Size:**	A total of 152 patients will be enrolled (76 in each group)
**Phase:**	III
**Number of Sites:**	At least 5 sites
**Description of Intervention:**	Two different blood pressure control strategies: conventional blood pressure control (CBP) versus augmented blood pressure (ABP) will be targeted based on study group assignment. The CBP group will have a target mean arterial pressure (MAP) of 65-70 mm Hg for 7 days after randomization or until ICU discharge; the ABP group will have a goal of MAP 85-90 mm Hg for 7 days after randomization or until ICU discharge.
**Study Duration:**	32 months
**Subject Participation Duration:**	Until completion of 6-month follow up
**Estimated Time to Complete Enrollment:**	24 months

Schematic of Study Design

Pre-Randomization

Perform pre-randomization assessments.
1) Screen potential subjects by inclusion and exclusion criteria; 2) Informed consent; 3) Demographic data; 4) Pre-randomization ASIA impairment scale; 5) Spinal column injury, imaging, and spinal procedures data; 6) Concomitant injuries and past medical history; 7) Pre-randomization vital signs.

Randomize
Complete Randomization Form

MAP 65-70
N subiects = 76

MAP 85-90
N subiects = 76

ICU & Hospital
Until H discharge

Perform ICU assessments.
1) ICU vital signs data (Days 1-7); 2) Sequential Organ Failure Assessment Score Form (SOFA) score; 3) Vasopressors and concomitant medications data; 4) Treatments non-compliance and discontinuation tracking; 5) Post 72 hour ASIA impairment scale; 6) ICU and hospital discharge.

Follow-Up
6 mo (+1 month)
post H discharge

Perform 6 month follow-up assessments.
1) Follow-up ASIA impairment scale; 2) International SCI Basic Pain Data Set; 3) Spinal Cord Independence Measure (SCIM); 4) Quality of life data; 5) Cardiovascular function data.

KEY ROLES AND CONTACT INFORMATION

**Principal Investigator:**	Miriam Treggiari, MD, PhD, MPH<br>Oregon Health & Science University<br>(503) 494-5735<br>treggiar@ohsu.edu
**Project Co-PI:**	David Zonies, MD, MPH<br>Oregon Health & Science University<br>(503) 494-5300<br>zonies@ohsu.edu
**Research Monitor:**	Peter Schulman, MD<br>Oregon Health & Science University<br>(503) 494-7641<br>schulman@ohsu.edu
**Project Biostatistician:**	N. David Yanez, PhD<br>Oregon Health & Science University<br>(503) 494-5354<br>yanezn@ohsu.edu
**Project Manager:**	Michael Kampp, BS, CCRP<br>Oregon Health & Science University<br>(503) 494-5224<br>kamppm@ohsu.edu
**Clinical Research Associate:**	Laura Sissons-Ross<br>Oregon Health & Science University<br>(503) 494-9545<br>sissonsr@ohsu.edu

1 ETHICS

1.1 Ethical Standard

This trial will be conducted according to the ethical principles that have their origin in the Declaration of Helsinki, good clinical practice (GCP), and applicable regulatory requirements. The investigator will ensure that this study is conducted in full conformity with the principles set forth in The Belmont Report: Ethical Principles and Guidelines for the Protection of Human Subjects of Research, as drafted by the US National Commission for the Protection of Human Subjects of Biomedical and Behavioral Research (April 18, 1979) and codified in 45 CFR Part 46 and/or the ICH E6.

1.2 Institutional Review Boards

The relevant institutional review boards (IRBs) for this trial will be provided with all documents required to fulfill their responsibilities. Any updates thereof will be provided according to GCP and applicable regulatory requirements. Trial activities will only start when approval from the relevant IRB is available. Documentation of all involved IRBs will be maintained according to GCP and applicable regulatory requirements.

1.3 Subject Information and Informed Consent

Before any trial-related procedure will be performed, freely given informed consent must be obtained. The informed consent discussion, the information sheet (if used), and the informed consent form provided to subjects will adhere to GCP and applicable regulatory requirements and will be approved by the relevant IRB prior to use. The informed consent discussion with the subject will be performed by the investigator or an appropriately trained delegate. The consent process will be documented in the clinical or research record. Subjects will be informed as soon as possible if new information becomes available that may be relevant to their willingness to continue participation in the trial. The communication of this information will be documented. Subject withdrawal of their consent for participation in the trial will also be documented.

1.4 Patient Confidentiality

In order In order to maintain patient privacy, data capture tools, study drug accountability records, study reports, and communications will identify the patient only by initials and the assigned patient number. The investigator will grant monitor(s) and auditor(s) from OHSU Coordinating Center or its designee and regulatory authorities access to the patient’s original medical records, including medical history, laboratory studies, and medication administrations, for verification of data gathered and to audit the data collection process. This information will be accessed for the duration of the research study, including the follow-up period, for the purpose of data reconciliation. The patient’s confidentiality will be maintained and will not be made publicly available to the extent permitted by the applicable laws and regulations.

1.5 Protocol Compliance

The Investigator will conduct the trial in compliance with the protocol provided by the Study Principal Investigators, and with approval/favorable opinion given by the IRB and the appropriate regulatory authorities. Modifications to the protocol may not be made without agreement of the Study Principal Investigators. Changes to the protocol will require a written IRB approval/favorable opinion prior to implementation, except when the modification is needed to eliminate an immediate hazard(s) to patients. The IRB may provide, if applicable regulatory authorities permit, expedited review and approval/favorable opinion for minor change(s) in ongoing trials that have the approval/favorable opinion of the IRB. OHSU Coordinating Center will submit all protocol modifications to the regulatory authorities in accordance with the governing regulations. When immediate deviation from the protocol is required to eliminate an immediate hazard(s) to patients, the investigator will contact the Study Principal Investigators at the OHSU Coordinating Center and/or the Research Monitor, if circumstances permit, to discuss the planned course of action. Any departures from the protocol must be fully documented on the appropriate case report form (CRF) and in the source documentation.

2 INVESTIGATORS AND TRIAL ADMINISTRATIVE STRUCTURE

2.1 Investigators and Trial Site Personnel

2.1.1 Investigators

The investigator, in general, is the person responsible for the conduct of the trial at the trial site and the safety of trial subjects. If the trial is conducted by a team of individuals at the trial site, the investigator leading and responsible for the team may be called the principal investigator (PI). All persons assigned responsibility as PI will be required to sign a declaration of their responsibilities before any trial-related procedure is performed. Curriculum vitae and/or other relevant documents confirming the qualifications of the investigator are required. This should include any previous training in the principles of GCP, experience obtained from work with clinical trials, and experience with subject care. Documentation of all involved investigators will be maintained according to GCP and applicable regulatory requirements.

2.1.2 Trial Site Personnel Assigned Trial-Related Duties

The investigators may define personnel at a trial site to perform significant trial-related procedures and/or to make trial-related decisions under his/her supervision. The investigator must maintain a signed list of appropriately qualified persons to whom he or she delegates significant trial-related duties/responsibilities; the delegated trial-related responsibilities must be specified in the list. When personnel or responsibility changes are made, the relevant documentation must be updated before any trial-related activities are performed. Documentation of all involved trial site personnel performing significant trial-related procedures and/or making trial-related decisions will be maintained according to GCP and applicable regulatory requirements.

2.2 Data Monitoring Committee

A Data and Safety Monitoring Board (DSMB) will periodically review safety information from the trial and monitor trial conduct and overall progress. The DSMB will have access to un-blinded treatment information to investigate unexpected safety findings of larger than expected differences in adverse events or other safety data between treatment arms. Procedures and management of the DSMB will follow the manual of operations (MOO) and standard operating procedures (SOPs) and will be documented in the DSMB Charter.

3 INTRODUCTION

3.1 Background Information

Systemic hypotension is a hallmark physiological response immediately following spinal cord injury (SCI). Prevention of hypotension (systolic blood pressure <90 mm Hg) appears to be an essential component of the early management of victims of SCI. While it is well documented that early aggressive management in an Intensive Care Unit (ICU) setting involving respiratory and cardiovascular support has been accompanied by considerable improvement in vital and functional outcomes in patients with SCI, the goals of hemodynamic resuscitation have not been investigated in a systematic manner.

The Congress of Neurological Surgeons recently published guidelines recommend maintenance of a mean arterial pressure (MAP) between 85 and 90 mm Hg for the first 7 days following the acute SCI.[1] However, this recommendation is only based on level III evidence from case series, non-experimental, and non-comparative studies.[2,3] These studies were not specifically designed to investigate the role of Targeted blood Pressure Management (TPM), rather were focused on process improvement for the overall management of patients with SCI. Considering the inadequate methodological quality, the generally small sample sizes, the lack of a comparison group, the uncontrolled conditions in the setting of concomitant therapies, and the non-contemporary overall management described in these historic study cohorts, there is an important need to provide high quality data from prospective trials that can be used to guide optimal hemodynamic care.

There is in theory a strong rationale for TPM to prevent secondary spinal cord insult. A series of animal based investigations performed in the 1970s and 1980s suggested that systemic hypotension after SCI was associated with reduced spinal cord perfusion and worse neurologic outcomes. Several case series in the subsequent decade suggested that maintaining a MAP >85 mmHg for 7 days was not associated with deleterious effects and may have provided benefit.

Of the major human studies, Levi et al studied SCI patients with a MAP >90 mm Hg to evaluate the role of invasive monitoring in the early management of such patients. Another hemodynamic study by Vale et al.[4] failed to show any clinical improvement with a MAP >85 mm Hg among patients who presented in shock. Taken together, these data call into question the validity of TPM and the basis of the proposed targets.

Targeted blood Pressure Management may require parenteral administration of vasopressor agents to maintain high MAP targets. Although considered safe in this patient population, high dose vasopressor support is not without consequence. While norepinephrine might have direct toxic effect on myocytes by inducing apoptosis,[5] phenylephrine use might be limited by the occurrence of reflex bradycardia.

The concept of TPM in neurological resuscitation assumes that the spinal cord blood flow is pressure passive when there is loss of autoregulation. However, not only the extent of impairment of spinal cord autoregulation is unknown, but also potent constrictors could potentially impair spinal cord blood flow by limiting collateral flow through intercostal anastomosis when increased pressure occurs at the expense of excessive vasoconstriction. The inability to measure or monitor spinal cord blood flow while managing blood pressure with potent vasoconstrictors, raises concerns regarding the beneficial effects of TPM.

3.2 Specific Aims / Study Design

The early critical care management of patients with SCI may have great influence over their inpatient morbidity and subsequent neurological prognosis. While the evidence supporting the avoidance of hypotension is strong, the role of TPM is much less well established. With clinical equipoise, it is critically important to determine the optimal targets for blood pressure management for cardiopulmonary support to maximize spinal cord perfusion. We propose a multi-center, randomized, controlled study to assess the efficacy and safety of early TPM in an ICU setting in patients with acute SCI. The study will equally randomize 152 acute SCI patients to either a MAP target between 65 and 70 mm Hg or MAP target between 85 and 90 mm Hg for a duration of 7 days or until ICU discharge. The randomization design will be unrestricted 1:1 or “fair-coin” design. We hypothesize that TPM, by enhancing spinal cord perfusion, will improve long term neurological and functional outcomes in patients with SCI. Our specific aims are as follows:

This multi-center, randomized, controlled study will assess the efficacy and safety of early TPM in an ICU setting in patients with acute SCI with blunt injury. The study will equally randomize 152 acute SCI patients to either Conventional Blood Pressure (CBP) with a MAP target between 65 and 70 mm Hg or Augmented Blood Pressure (ABP) with a MAP target between 85 and 90 mm Hg for a duration of 7 days or until ICU discharge. The study will include patients with acute SCI of American Spinal Injury Association (ASIA) grade A, B, or C involving the cervical or thoracic spine as documented by a combination of clinical exam and Computerized Axial Tomography (CT) or Magnetic Resonance Imaging (MRI) findings of cervical or thoracic abnormalities consistent with the clinical findings. The study will test the overall hypothesis that ABP will improve long term neurological and functional outcomes in patients with SCI by maintaining adequate spinal cord perfusion.

3.3 Potential Risks and Benefits

3.3.1 Potential Risks

Risks associated TPM include: pulmonary edema, generalized edema, and cerebral edema for volume replacement and increased cardiac work, cardiac ischemia, bradycardia, and organ hypoperfusion from the administration of vasopressors or inotropes for blood pressure manipulations. These treatment strategies are widely utilized in clinical practice with presumed significant potential benefits and the risks of the research do not exceed current usual care;

therefore, the ethical principles of beneficence and non-maleficence are respected.Though there is no invasive monitoring requirement, hemodynamic management may require invasive monitoring with the risk associated with placement and presence of indwelling catheters as dictated by clinical needs. Additionally, the patients who have been randomized in the study will undergo cerebral MRI and/or spine CT as part of standard of care imaging for SCI and may suffer from claustrophobia and anxiety due to the tight configuration of the apparatus.

Additional laboratory work is not required for study purposes.

There is always a risk of breach of confidentiality when accessing patients' records. However, every necessary precaution will be taken to minimize such risk.

3.3.2 Frequency of TPM Associated Risks

Less likely (happen in less than 20% of people)

- pulmonary and generalized edema
- cardiac ischemia
- organ hypoperfusion

Rare (happen in less than 3% of people)

- cerebral edema
- bradycardia

3.3.3 Mitigation of Potential Risks

As the management strategies proposed are within the range of current clinical practice in this population, there is no anticipated incremental risk associated with the participant assignment to any of the proposed treatment strategies. Therapies will be implemented by experienced and certified personnel, familiar with the clinical monitoring and the proposed hemodynamic management strategies. Therefore, the risks associated with TPM, as proposed in this protocol, do not exceed the risks taken as part of current routine patient care.

Particular attention has been paid in the designing of the treatment strategies to prevent treatment misalignment. All patients in the two pressure arms will receive treatment, so that no participant will be precluded of appropriate management. Likewise, participants with lower hemodynamic goals should not experience under-treatment, as all participants in both groups are receiving treatment, allowing for treatment intensity increases.

As added protection for study participants, the protocol is designed so that per-protocol treatments will be discontinued in the event the participant develops worsening neurological symptoms. In such cases, additional therapy escalation will be allowed for temporary periods when a blood pressure challenge might be transiently clinically indicated. Patients will continue to receive standard clinical care from their physician.

3.3.4 Potential Benefits

Potential benefits to subjects enrolling in this trial may include a greater recovery of neurological symptoms, allowing to achieve higher level of functioning and performance as assessed by a neurological evaluation, functional independence measures, and quality of life assessment at six months, as well as benefits of medical supervision from being in a clinical trial.

Improvement of lack of symptoms progression may occur following either treatment arm. Patients randomized to the higher MAP will benefit by adhering to the current guideline for the management of acute spinal cord injury issued by the Consortium for Spinal Cord Medicine who recommend maintaining a MAP target of 85-90 mm Hg for seven days after the injury. The rationale of the guidelines is that higher MAP might maintain better perfusion to the spinal cord and improve long term neurological function. Therefore, there is potential for better neurological outcome, higher functioning and improved quality of life in this group. However, this is hypothetical and it has not been demonstrated. Patients randomized to the lower MAP will benefit from lower doses of vasopressors which have associated risks of excess fluid in the lungs, swelling of the brain, general swelling, heart attack, and inadequate oxygen supply. Future patients might benefit if the lower MAP approach was found not result in worse long term neurological function.

All these outcome benefits will result in a reduction in institutional and societal costs and disability burden. These benefits are particularly meaningful as this disease frequently affects young, previously healthy individuals, who are typically key contributing members of society. Indirect benefits will be shared by family members and society as a whole.

4 OBJECTIVES

4.1 Study Objectives

The primary hypothesis for the current study is that ABP will enhance spinal perfusion pressure, prevent hypoperfusion and improve preservation of spinal cord viability in areas suffering from ischemia, hemorrhage or edema. Preservation of adequate spinal cord perfusion pressure may result in clinical benefits, as indicated by at least the equivalent of a 5 point difference in the ASIA motor or sensory scores at six months. The study is designed to prove that the study treatments, when started in the immediate post-injury period, work well to prevent secondary injury and to attenuate its deleterious effects. If the study intervention does not have an impact on outcome, then these findings would indicate that there is no need to provide hemodynamic augmentation after acute SCI other than preventing hypotension.

4.2 Study Outcome Measures

4.2.1 Primary Specific Aim

The primary aim of this study is to determine the differential effects of two levels of MAP on long-term motor and sensory outcomes after acute SCI. Compared to maintaining a MAP target between 65 and 70 mm Hg, maintaining a MAP target between 85 and 90 mm Hg for a duration of seven days or until ICU discharge may improve motor and sensory ASIA scores at six months relative to baseline.

4.2.2 Secondary Specific Aim for Efficacy

The secondary specific aim for efficacy is to determine the differential efficacies of two levels of MAP based on long-term pain and functional independence measures. Compared to maintaining a MAP target between 65 and 70 mm Hg, maintaining a MAP target between 85 and 90 mm Hg for a duration of seven days or until ICU discharge may improve functional outcome measures at six months on the Spinal Cord Independence Measure III, the International Spinal Cord Injury Basic Pain Data Set, Quality of Life and Cardiovascular Function.

4.2.3 Specific Aims for Safety

The specific aim for safety is to determine the differential safety profiles of maintaining two different levels of MAP for seven days or until ICU discharge. Compared to maintaining a MAP target between 65 and 70 mm Hg, maintaining a MAP target between 85 and 90 mm Hg for a duration of seven days or until ICU discharge may have a more favorable safety profile as measured by respiratory complications (a composite of acute respiratory distress syndrome, hypoxemia as indicated by PaO_2/FiO_2 ratio, and pneumonia), occurrence of myocardial ischemia or infarction (troponin levels), maximum organ dysfunction as measured by the Sequential Multiple Organ Failure score (SOFA) in the first seven days after injury or until ICU discharge - whichever comes first -, duration of mechanical ventilation, and hospital stay.

5 STUDY DESIGN

This Phase III multicenter, randomized, controlled study will investigate the efficacy and safety of a strategy consisting of ABP to a MAP of 85-90 mm Hg compared to a strategy of CBP with a MAP goal of 65-70 mm Hg in patients suffering from acute SCI.

All patient volume status will be managed with fluid administration with the goal of maintaining euvolemia. Approximately 152 patients will be randomized to detect the main effects of TPM. Investigators will make the best effort to randomize and start study procedures within 24 hours, however, longer delays can be considered up to a 48 hour window. Prior to randomization, the patient ASIA status will be recorded. For study purposes, ASIA score documentation will be repeated between 72 hours after randomization and prior to hospital discharge, and then at six months post-randomization.

After obtaining informed consent from the participant or legally authorized representative (LAR), subjects will be randomly allocated to one of the two treatment arms: CBP and ABP goals. A computerized randomization system will verify eligibility and randomly assign eligible participants to one of the two different study arms. Randomization will be performed using an unrestricted or “fair-coin” design; participants will be allocated 1:1 to the two treatment groups. The group assignment will remain the same throughout the study duration.

The TPM protocol will be maintained for seven days or until ICU discharge. Vital signs and urine output will be monitored hourly during seven days or until ICU discharge. There will be daily monitoring of hemodynamic data, fluid intake/output and fluid balance. Subjects will be evaluated at 6 months after SCI for complete ASIA score assessment, evaluation of pain levels using the basic pain dataset, functional independence measures, quality of life, and assessment cardiovascular status.

5.1 Setting

The study coordinating center will be located at Oregon Health & Science University (OHSU) in Portland, Oregon. This will be a multi-center trial where OHSU will act as both the coordinating center and as a participating site.

STUDY ENROLLMENT AND WITHDRAWAL

5.2 Subject Enrollment Procedure

Before the informed consent is signed, patients will be screened to identify subjects who could potentially be enrolled into the trial. Potentially eligible subjects or their LAR will be asked by the investigator to enroll into the trial by giving written informed consent. The investigator will keep a subject screening log and a subject identification and enrollment log.

5.3 Inclusion/ Exclusion Criteria

Subjects eligible to participate in the trial will be adult patients of either gender, admitted to any of the participating centers for the treatment of acute SCI. Subjects who meet all inclusion criteria and no exclusion criteria will be considered for enrollment in this clinical investigation.

5.3.1 Inclusion Criteria

**Clinical Inclusion Criteria**	IC1.	Acute traumatic SCI involving neurological levels as defined by the ASIA neurological examination between C0 and T8 (tetraplegia) and resulting in new onset neurological deficits consistent with an ASIA motor assessment of level A, B, or C.
	IC2.	18 years of age or older.
	IC3.	Signed consent by study participant or applicable LAR.

5.3.2 Exclusion Criteria

**Clinical Exclusion Criteria**	EC1.	Penetrating SCI injury.
	EC2.	Isolated cauda equine syndrome or injury at bony level Th9 or below.
	EC3.	Pre-existing motor deficit secondary to chronic myelopathy.
	EC4.	History of demyelinating disease or central nervous system autoimmune disorder.
	EC5.	History within the past six months and/or physical findings on admission of decompensated congestive heart failure (NYHA functional class III or IV, or objective class C or D).
	EC6.	Acute, evolving or recent (30 days) myocardial infarction.
	EC7.	Chronic renal failure requiring dialysis.
	EC8.	Suspected or confirmed pregnancy.
	EC9.	Severe terminal disease with life expectancy less than 6 months.
	EC10.	Severe traumatic brain injury at presentation (GCS ≤8) with confirmation of injury on brain imaging.
	EC11.	A condition that would preclude the performance of an accurate neurological exam due to a prior diagnosis of Alzheimer's disease, stroke, degenerative condition, cerebral tumor, or mental retardation.
	EC12.	Non-English or Non-Spanish Speaking.
	EC13.	Refusal of consent.

5.3.3 Inclusion of Women and Minorities

There will not be exclusions for women or minorities and recruitment of these populations will be monitored by the OHSU Coordinating Center. Women will be included in this study in proportion to their representation in the target population. Female and male subjects will be recruited equally for participation in this study. Only women with a positive pregnancy test will be excluded in order to prevent exposure to any potential incremental risks that could be associated with participation in a research trial in the absence of known benefits.

All planned study sites serve diverse multi-ethnic populations. To ensure adequate enrollment of minorities we will conduct the neurological assessment of Spanish speakers with the assistance of Spanish translators. Since the neurological exam depends on patient's participation, groups speaking other languages than English or Spanish will not be included because it would not

possible to reliably find in-person translators available to assist with the clinical evaluation in other languages.

5.3.4 Exclusion of Vulnerable Populations

The following populations will be excluded from the study: children, pregnant women, prisoners, and decisionally impaired adults for whom a LAR is not available.

5.4 Strategies for Recruitment

A research coordinator will review daily the medical records of neurosurgical patients admitted to ICUs at any of the participating clinical sites for study eligibility of their patient population. Use of the medical records for review of data for study eligibility will be performed in accordance with institutional, state and Health Insurance Portability and Accountability Act (HIPAA) regulation.

Patients suffering from acute SCI admitted to one of the participating centers will be prospectively identified. Potential study subjects will be identified from patients presenting to the Emergency Department or directly admitted to the Trauma/Surgical or Neurocritical Care Units at the participating sites. Patients who have a documented acute SCI based on clinical findings and radiological abnormalities on CT or MRI of either vertebral fractures, ligamentous injury, disc herniation, or spinal cord contusion, compression, edema, or infarct will be immediately evaluated by the on-call attending physician with documentation of the ASIA score and evaluated for eligibility as soon as possible after presentation.

A de-identified log of patients not approached within 48 hours of hospital admission or refusing participation in the study will be maintained to define the characteristics of this population. If a non-enrolled patient refuses to allow the investigator to access the medical record, no data will be maintained. All procedures for obtaining consent will be approved by the institutional human subjects review board.

5.5 Informed Consent Process

Adults 18 years or older with an acute traumatic SCI involving neurological levels as defined by the ASIA neurological examination between C0 and T8 (tetraplegia) and resulting in new onset neurological deficits consistent with an ASIA motor assessment of level A, B, or C will be eligible for participation in the study if approached within 48 hours of hospital admission.

Potential study subjects/LAR will be able to learn about the study in a variety of ways. They may be approached after admission to the ICU by the study Research Coordinator, who will provide study documentation approved by the IRB, which will discuss the purpose of the study and the procedures involved. They may also learn about the clinical trial via the clinical trial website or be given information about the study by a critical care physician or certified nurse while staying in the ICU.

If a patient/LAR expresses interest in participation in the trial, the Research Coordinator will provide further information regarding the objectives of the study, study requirements, procedures and the potential risks.

All protocols will require that all study participants or a member of a patient's family sign and date an IRB approved informed consent form. Many patients may not be able to grant consent during the window required for enrollment in the study; therefore, their family members will receive the information about the study and asked to sign the informed consent form. However, the patient will be notified and asked for verbal consent for continued participation as soon as the participant regains legal competency. If the study participant is legally competent but is physically unable to talk or write, an unbiased witness who is neither a study team member nor a family member of the participant can sign the witnessed line on the consent form to enter the patient into the trial. The unbiased witness must observe the consent process.

5.6 Point of Enrollment

A subject will be considered enrolled in the study at the time that the informed consent form is signed. Subjects who are discontinued prior to randomization are considered "enrolled but not randomized." Only randomized subjects will be included in the study analysis.

5.6.1 Subject Trial Cards

Subjects who are randomized in the trial will receive a trial card. The trial card will list the following information: 1) Name of the subject and a statement that he/she is currently participating in a clinical trial; 2) Trial code; 3) Dates of all individual visits; 4) Name of investigator; and 5) Contact telephone number at the trial site. The card will be collected at the subject's last trial visit.

5.6.2 Subject Retention

Once a participant enrolls in this study, the study site will make every effort to retain him/her for six months of follow-up to preserve the integrity of the study and to minimize possible bias associated with loss due to dropouts. To enhance participant retention, patients or their LAR will be adequately educated during the informed consent process. Study staff will be trained to explain to the participant or their LAR the importance of the follow-up visit and the scientific relevance of their data for the study and the potential deleterious effect that missing data could on the trial's integrity and credibility. Appropriately worded informed consent forms will enable patients to make more informed decisions about their willingness to join the trial and to participate in continued follow-up without feeling pressured to doing so.

At time of enrollment the research coordinator will collect the participants' mailing address, contact number, email address as well as contact information for an emergency contact who will know how to get in touch with subject, as well as the primary care physician information. A clinical medical records release form will also be obtained to allow review of the patient's outside medical

record for ASIA assessment and cardiovascular data related to the 6-month follow-up visit. The study coordinator will initiate follow-up contact with the patient beginning four to six weeks prior to the six month mark from the patient's enrollment. The study coordinator will attempt to contact the patient by telephone or email. Should the patient not respond within two weeks, the study coordinator will call the patient's emergency contact and request a current phone number for the patient. Contact attempts will continue for four additional weeks. If the patient still has not responded he or she will be considered lost to follow-up. For patients who are able to be reached, a window of six weeks beginning two weeks prior to the six month mark and continuing four weeks after will be suitable for conducting follow-up.

For participants unable or unwilling to return to the study site for the six month follow-up visit, research study coordinators will be available to travel to the participant's residence to conduct the assessment. All participants will be contacted for follow-up, including participants that did not complete study treatment, unless they have formally withdrawn consent.

5.7 Treatment Assignment Procedures

5.7.1 Randomization Procedures

Subjects should be approached as soon as possible after hospital admission and will be randomized within 48 hours of being approached for study participation to allow for adequate time for patient identification, the informed consent process, patient enrollment, and collection of baseline variables. Subjects who meet the inclusion and exclusion criteria and consent to the study will be assigned to one of two study groups using a computer-based randomization procedure. The randomization procedure will require confirmation of the eligibility criteria and ensure that the integrity of the randomization is maintained. Subjects will be randomized to a treatment arm based on an unrestricted or "fair-coin" randomization procedure. The randomization will be carried out in SAS using proc plan with a specific random number seed so that the results are reproducible. This list will be used at clinic sites to randomize subjects as they enroll in the study. Subject randomization assignments will only be accessible to the bioanalytical staff assigned to the project who have been designated to be unblinded.

5.7.2 Blinding

This is an assessor-blinded study. Due to the requirement for targeting specific blood pressure goals, the primary caregivers (attending physician and nurse in charge of the patient) will not be blinded to the treatment allocation. In order to maintain blinding, a baseline neurological exam will be conducted and ASIA score will be documented immediately prior to obtaining informed consent and randomization assignment. Subjects will not be notified of the treatment arm assignment and he/she will be unlikely to identify which treatment has been administered. In order to ascertain whether subject blinding was maintained, the subject will be asked whether he/she learned the subject's group assignment at the end of follow-up assessment. The outcome examiner that will collect the 6-month study endpoints will be blinded to treatment assignment. The DSMB will

remain blinded to treatment assignment when outcome data is reported. The treatment assignment groups will be reported as group A and group B.

5.8 Subject Discontinuation

5.8.1 Investigator Initiated Withdrawal

There are no compulsorily reasons to withdraw a patient from the study unless, in the clinical opinion of the investigators, continuing participation in the study represents an excessively high risk for the study participant. Investigators are reminded that it is important to minimize missing data for safety and efficacy evaluation; therefore, subjects should be kept in the trial unless medically necessary.

5.8.2 Subject Initiated Withdrawal

While study withdrawal is discouraged, subjects may withdraw from the study at any time, with or without reason and without prejudice to further treatment. In all cases of withdrawal or discontinuation, the investigator will make all reasonable efforts to determine the reason for the subject's withdrawal. All subjects enrolled in the clinical study shall be accounted for and documented. If such withdrawal is due to problems related to the investigation, the investigator shall ask for the subject's permission to follow his/her status or condition outside of the clinical study.

5.8.3 Procedures for Handling Discontinued Subjects

The investigator must document any discontinuation of a subject. Where applicable, the relevant IRB must also be informed with a detailed written explanation. The following must be done for all discontinued subjects: 1) Document reason for discontinuation; 2) Document status of all new and ongoing (serious) adverse events; 3) Document all demographic data, including the date of the signature on the informed consent form; and 4) Complete any other trial-related formalities.

5.9 Premature Termination or Suspension of Study

The relevant IRB, the regulatory authorities, or the PI or assigned delegate alone or in conjunction have the power to make a binding decision to prematurely terminate or suspend the trial at any or all trial sites. The DSMB also may recommend termination of the trial. In addition, for an individual trial site, this decision can be made by the PI. The party prematurely terminating or suspending the trial must promptly inform all other parties (i.e., the PI(s), the relevant IRB, the relevant regulatory authorities, or OHSU Coordinating Center or assigned delegate, as applicable).

In addition, if the investigator decides to terminate or suspend the trial at the trial site, the investigator must promptly inform the subjects, ensure appropriate follow-up for any enrolled

subjects, and provide the relevant IRB, OHSU Coordinating Center or assigned delegate as applicable, with a written explanation of the termination or suspension.

6 STUDY INTERVENTION

6.1 Study Treatment Protocols

After obtaining informed consent, acute SCI participants will be equally randomized to be maintained on two different blood pressure targets for seven days or until ICU discharge. Participants will be monitored by the critical care team during the ICU stay. Patients will be treated with standardized volume replacement therapy with crystalloids with the goal of maintaining euvolemia. Vasopressors, including phenylephrine, norepinephrine, vasopressin, dopamine, or a combination of these agents as per standard of care at each participating institution, will be used to maintain blood pressure within the specified targets. Participants will have their standard of care vasopressors titrated to a target MAP of 85-90 mm Hg for seven days or until ICU discharge (as recommended in the guidelines for the hemodynamic management of acute SCI) or to a target MAP of 65-70 mm Hg for seven days or until ICU discharge (considered to be a general standard MAP aiming at preventing hypotension). It is anticipated that the high blood pressure target group will be exposed to vasopressors more frequently, in higher doses and for a longer duration.

After randomization, the nurse in charge of the patient will implement the fluid and pressure protocol according to assigned treatment. Patients will be maintained in the same treatment arm to which they were initially assigned throughout the duration of the study. Overlapping of the therapeutic goals across each assignment group will be avoided to ensure adequate distinction among the treatment arms.

The hemodynamic assessment used to guide the pressure management protocol will be obtained at least every four hours for all patients. This includes blood pressures and vital signs, and recording of central venous pressure, if available. From these data, the appropriate action (initiation or continuation of pressors, or anti-hypertensives) will be implemented. If, prior to the next scheduled 4-hour assessment, a change in one of the monitored parameters occurs (e.g., decrease in blood pressure), then the appropriate action can be taken earlier. If a study participant becomes hypertensive during the course of the study period, his/her home anti-hypertensive should be restarted in the conventional blood pressure group and can be restarted in the augmented blood pressure group, as clinically indicated. In the case of hypertension, clinical management should be directed to maintain participants in their blood pressure assignment range.

To evaluate appropriateness of organ perfusion, we will also collect daily urine output and fluid balance, and clinical assessment of the effectiveness of the arterial circulation, using the organ system listed in the Sequential Organ Failure Assessment score, and troponin values.

Detailed management algorithms with instructions for systematic adjustment to allow immediate adaptation to the evolving clinical course. The treatment algorithms are outlined in APPENDIX B.

6.1.1 Targeted blood Pressure Management

Immediately after randomization, two different blood pressure control strategies: CBP versus ABP will be targeted based on study group assignment. The CBP group will have a target MAP of 65-70 mm Hg for seven days after randomization or until ICU discharge, whichever comes first; the ABP group will have a goal of MAP 85-90 mm Hg for seven days after randomization or until ICU discharge, whichever comes first. The blood pressure targets will be achieved by the administration of vasoactive medications (vasopressors or antihypertensives) to maintain the MAP within the target range.

6.1.2 Volume Expansion to Achieve Euvolemia

Volume resuscitation will be achieved according to standard of care at each participating institution. The standardized fluid administration strategies will be initiated in all patients with the goal of maintaining normovolemia. Depending on local standard of care, normovolemia will be based on a combination of hemodynamic and laboratory parameters including daily fluid balance, lactate and base deficit, and/or central venous pressures (CVP) if a central venous catheter is in place. It is expected that for typical maintenance of normovolemia the administration of intravenous or enteral fluid replacement approximate 30 mL/kg/day.

6.2 Assessment of Clinicians' Compliance with Study Procedural Intervention

Compliance with protocol instructions will be monitored daily, preferably during a morning reference period and again at a randomly selected time. A 100 percent audit of all instructions will be conducted for the first 5 enrolled patients at each site to ensure high protocol compliance; random checks will be conducted subsequently.

Non-compliance with the study algorithm should be discouraged at all times. If for any reasons the patient blood pressure needed to be altered outside the ranges of study algorithms, a temporary blood pressure challenge will be documented in the treatment non-compliance log of the case report form, specifying new target of augmentation, the duration of the challenge, and the reason why this was necessary. Patients should be returned to their assigned randomization group and follow the per-protocol instructions at the earliest possible time. If a treatment needed to be permanently and prematurely discontinued, then the time and reason for discontinuation should be documented and the event should be reported within 24 hours to the OHSU Coordinating Center.

We will track each event of blood pressure non-compliance using a designated event tracking form that documents the time of onset and termination of the out-of-range period, its duration, and the new target levels.

We define treatment failure as the requirement for permanent discontinuation of the assigned treatment algorithms.

7 STUDY SCHEDULE AND EVALUATIONS

7.1 Pre-randomization Assessment

Written informed consent must be obtained for all patients who are potential study candidates. Inclusion and Exclusion criteria listed above respectively, will be assessed to determine study candidate eligibility. Subjects who have provided informed consent and who have been determined to not meet all eligibility requirements will be withdrawn. Study candidates must sign the informed consent form before any study-specific tests or procedures are performed.

Pre-randomization assessments will be performed immediately prior to randomization and include: 1) Date and time of symptom onset; 2) Demographic Data (sex, race/ethnicity, age, height, weight); 3) Neurological exam (ASIA Assessment) with documentation of SCI severity and level and mechanism of injury; 4) Neuroimaging findings from spine CT or MRI Clinical ICU Assessment; 5) Spinal intervention and spinal procedures data; 6) Concomitant injuries and past medical history, in particular history related to hypertension and respiratory issues; 7) Injury severity score; 8) Monitoring of pre-randomization vital signs (Non-invasive BP and MAP with indication of the location of measurement, e.g., either the left upper extremity or the right upper extremity); 9) Mechanical ventilation; 10) Fluid Intake/Output; 11) Current vasopressors.

7.2 ICU Visit Assessment

All subjects will be monitored in the ICU or acute care for hemodynamic vital signs, e.g., heart rate (HR) and MAP starting at screening (baseline), prior to initiating the study protocols, and every 4 hours thereafter until study day 7 or ICU discharge. Non-invasive blood pressure and MAP will be recorded from the same side of the body to minimize measurement error. Central venous catheterization or other types of invasive monitoring will not be required for study purposes, but the decision will be left to the discretion of the treating physicians. Likewise, invasive arterial blood pressure monitoring will not be required for study purposes and it will be initiated based on clinical indication. If a study participant is discharged from the ICU or the intermediate care unit to the general ward prior to day 7 after inclusion in the study, the study hemodynamic goals may be continued using enteral vasoactive agents, e.g. midodrine or ephedrine. Vital signs, physiologic variables including systemic pressure and HR will be monitored frequently and at least every four hours while the patient is in the ICU, and until study day 7. Complications will be collected until hospital discharge.

7.2.1 Concomitant Treatments and Supportive Care

All participants should be given appropriate supportive therapy during the course of the study. During the hemodynamic management protocols, all the vasoactive agents administered parenterally or internally will be collected. For the parenteral administration, vasopressor agents will be converted to norepinephrine equivalents using the conversion table below.

Vasopressors Conversion Table		
**Agent**	**Dose**	**Norepinephrine equivalent**
Epinephrine	0.1 mcg/kg/min	0.1 mcg/kg/min
Norepinephrine	0.1 mcg/kg/min	0.1 mcg/kg/min
Dopamine	15 mcg/kg/min	0.1 mcg/kg/min
Phenylephrine	1.0 mcg/kg/min	0.1 mcg/kg/min
Vasopressin	0.04 U/min	0.1 mcg/kg/min

During the time of study and in addition to the pressure treatment strategies provided according to the study protocol, associated treatments for the protection of the spinal cord and overall care will be recorded. These include steroid class agents. These medication classes that are administered during the study will be recorded on the appropriate CRF along with the start and stop date(s), dose and amount administered, and indication. Steroids will be converted to methylprednisolone equivalents using the conversion table below.

Corticosteroid Conversion Table		
**Glucocorticoid**	**Approximate Equivalent Dose (mg)**	**Half-life (hours)**
**Short-Acting**		
Cortisone	25	8-12
Hydrocortisone	20	8-12
**Intermediate-Acting**		
Methylprednisolone	4	8-36
Prednisolone	5	8-36
Prednisone	5	8-36
Triamcinolone	4	8-36
**Long-Acting**		
Betamethasone	0.6-0.75	36-54
Dexamethasone	0.75	36-54

7.2.2 Documentation of post-72 hour ASIA Score

Between 72 hours after the injury and prior to ICU discharge, a complete ASIA assessment will be repeated to document the post-resuscitation ASIA score.

7.2.3 Hospital Discharge

The duration of hospitalization, duration of mechanical ventilation, and discharge disposition will be recorded at the time of hospital discharge and transfer to inpatient or outpatient rehabilitation.

7.2.4 Adverse Event Monitoring

In the case of an adverse event (AE), the subject will be treated by the primary physician or referred for care as appropriate. Data pertaining to any AE will be entered into the CRF. For any AEs, a causal or symptomatic treatment according to standard medical practice will be performed if deemed necessary by the investigator. The medical care given to and medical decision made on behalf of the subjects will always be the responsibility of a qualified physician.

7.3 Six Month Follow-Up Assessment

Patients will be evaluated at 6 months (+1month) after SCI given the time required for neurological recovery and stabilization of their clinical condition. A complete ASIA score assessment, evaluation of pain levels, independence measures, quality of life, and cardiovascular function data will be collected during an in-person visit. For sites that routinely have 6-month follow up visits, the study visit will be arranged in conjunction with their scheduled clinic visit. By six months, the vast majority of the patients should attain a level of recovery that approximates their final condition. Every effort will be made to collect 6-month data for all study outcomes to best insure the integrity of randomization and the trial results. The only two valid reasons a patient can be off study are: 1) withdrawal of consent or 2) the achievement of all required efficacy and safety end point information. All reasonable efforts should be made to ensure patients who go off study treatment are consistently followed for outcomes unless they withdraw consent.

7.3.1 Patient Tracking at Follow-Up

There is high mortality in this population. We will capture deaths among participants lost to follow up, using various mechanisms including review of the medical record, contacting the legal next of kin or the contact provided at the time of study enrollment. For patients who are lost to follow up, we will also contact the primary care physicians and safety net clinics to ascertain the participant's vital status. In addition, local obituaries and newspapers will be screened for potential identification of lost to follow-up study participants.

8 ASSESSMENT OF SAFETY

8.1 Specification of Safety Parameters

The following safety data will be collected during the trial: AEs and vital signs. Clinically relevant abnormal values, based on investigator judgment, will be recorded as AEs.

8.1.1 Unanticipated Problems

Unanticipated problems include any incident, experience, or outcome that meets all of the following criteria:

- Unexpected in terms of nature, severity, or frequency give 1) the research procedures that are described in the protocol and related documents; and 2) the characteristics of the subject population being studied;
- Related or possibly related to participation in the research; and
- Suggests that the research places subjects or others at a greater risk of harm, including physical, psychological, economic, or social harm, than was previously known or recognized.

Incidents, experiences, or outcomes meeting all three criteria above will warrant corrective actions in order to protect the safety, welfare, and rights of subjects.

8.1.2 Protocol Deviation

A protocol deviation is any accidental or unintentional change to, or non-compliance with the research protocol that does not increase risk or decrease benefit; does not have a significant effect on the subject's rights, safety or welfare; and/or the integrity of the data. A deviation may be due to the research subject's non-compliance, or an unintentional change to or non-compliance with the research protocol on the part of a researcher. When a protocol deviation is discovered, it is the PI's responsibility to assess all protocol deviations.

Protocol deviations must be submitted to the Study PIs at the OHSU Coordinating Center or designated authority within 10 days of first knowledge of the investigator using the Protocol Deviation Form and must include: 1) Detailed narrative describing the deviation, how the deviation was discovered, the risks the subjects were exposed to and the measures taken to minimize risk; 2) A detailed corrective action plan to prevent similar deviations in the future.

Protocol deviations must be tracked in a protocol deviation log. Protocol deviation logs should be submitted at the time of continuing review. Research teams should review the protocol deviation logs periodically and determine if the deviations indicate a larger systemic problem with the

implementation of the research. Appropriate corrective measures should be taken to rectify any systemic problems.

8.1.3 Adverse Events

An AE is any untoward medical occurrence in a subject enrolled in a clinical trial. An AE can therefore be any unfavorable or unintended sign, symptom, or disease temporally associated with the trial procedures. Pre-existing diseases or conditions occurring before enrollment are not considered to be AEs unless there is an untoward change in intensity, frequency, or quality after enrollment. Lack of efficacy is not considered to be an AE.

8.1.4 Serious Adverse Events

A serious adverse event (SAE) is any untoward medical occurrence that: 1) Results in death; 2) Is life-threatening; 3) Requires inpatient hospitalization or prolongation of existing hospitalization; 4) Results in persistent or significant disability or incapacity; 5) Is a congenital anomaly or birth defect; or 6) Is considered a clinically important medical event.

An elective hospital admission (e.g., for pre-planned surgery) will not be considered a SAE if documented at enrollment. Short-lasting (<24 hours) hospital admissions (e.g., for clinical check-ups) not meeting any of the other above mentioned criteria will also not be considered SAEs.

8.2 Characteristics of an Adverse Event

8.2.1 Relationship to Study Intervention

The causal relationship of an AE to the study protocol will be classified using the following terminology. The given criteria for each term are neither exhaustive nor required to be fulfilled in total for the selection of the respective term:

**Relationship to Study Intervention**	Conditional/ Unclassified	Additional data for proper assessment are under examination.
	Not Classifiable	The available data cannot be judged because information is insufficient or contradictory, and cannot be supplemented or verified.
	Not Related	Data with sufficient evidence to accept that there is no causal relationship.
	Unlikely	Data without sufficient evidence to accept that there is no causal relationship, but also with no evidence or argument to suggest a causal relationship.

**Relationship to Study Intervention (cont.)**	Possible	Data with limited evidence or argument to suggest a causal relationship.
	Probable/ Likely	Data with sufficient evidence or argument to suggest a causal relationship.
	Certain	Data with clear evidence for a causal relationship.

8.2.2 Expectedness of SAEs

Expectedness of (serious) adverse events will be assessed by the OHSU Coordinating Center. An unexpected AE is one where the nature or intensity is not consistent with available information. Furthermore, reports that add significant information about the specificity or severity of a known, already documented, adverse reaction constitute unexpected AEs. For example, an AE that is more specific or more severe than expected would be considered “unexpected”.

8.2.3 Severity of Event

The clinical intensity of an unanticipated problem or AE will be classified as described below. For problems or events where the intensity changes over time, the maximum intensity observed during the whole duration will be documented.

**Severity of Event**	Mild	Signs and symptoms that can be easily tolerated, ignored, and disappear when the subject is distracted.
	Moderate	Symptoms cause discomfort but are not tolerable, cannot be ignored, and affect concentration.
	Severe	Symptoms affect usual daily activity.

8.2.4 Outcome of Event

The outcome at the time of last observation will be classified as: 1) Recovered/Resolved; 2) Recovering/Resolving; 3) Recovered/Resolved with sequelae; 4) Not Recovered/Not Resolved; 5) Fatal; or 6) Unknown.

8.3 Time Period and Frequency for Event Assessment and Follow-Up

All AEs will be documented from the time of enrollment (i.e., the time the informed consent form is signed) up to the time of the last protocol scheduled contact has occurred (i.e., date of last visit/contact, including phone call). Subjects will be monitored daily for unanticipated problems and AEs for the duration of the study. Any unexpected problem, unanticipated problem, or clinically relevant abnormal laboratory or vital sign result will be followed until it reaches a

satisfactory conclusion, becomes stable, or clinical judgment indicates that further evaluation is not warranted.

8.4 Reporting Procedures

For the duration of patient therapy, the investigators will be responsible for monitoring and recording AEs related to vasopressors related side effects, new neurological event or new or worsening organ dysfunction. When possible, severity of AEs will also be recorded. Possible AEs due to treatment include pulmonary edema, cardiac or other organ ischemia, organ dysfunction or failure, and arrhythmia and will be monitored during treatment. Study site investigators will also be responsible for recording unanticipated AEs observed by patients or investigators.

Subjects will be questioned about possible unanticipated problems and AEs with non-leading questions daily. All unanticipated problems and AEs reported spontaneously by subjects at any time point will also be documented. All unanticipated problems and AEs will be documented with the following information, as appropriate: 1) Description (verbatim term); 2) Start date/time; 3) End date/time or continuation; 4) Relationship to intervention; 5) Seriousness; 6) Intensity; 7) Outcome; and 8) Action taken.

8.4.1 Unanticipated Problem Reporting

Federal regulations require prompt reporting of unanticipated problems and AEs to the IRB, appropriate officials, the funding agency or coordinating center, and the federal departments or agency heads as applicable.

**Unanticipated Problem Reporting**	Unanticipated Problem	Report within 5 working days of the researcher becoming aware of the event.
	Internal Adverse Event	Report within 5 working days of the researcher becoming aware of the event.
	External Adverse Event	Report within 10 working days of the researcher becoming aware of the event.
	Any permanent or temporary hold on some or all research activities.	Report within 10 working days of the researcher becoming aware of the event.

8.4.2 Serious Adverse Event Reporting

All SAEs (including death, irrespective of cause) must be reported to the OHSU Coordinating Center as soon as possible and no later than 24 hours after learning of the event. The trial site will be provided with contact details for these personnel before any trial related procedure is performed. The investigator must submit a safety reporting form, which includes a description of

the event, therapy instituted, and trial procedures. The following information must be communicated with the first notification of a SAE: 1) Trial identifier; 2) Subject identifier; 3) Subject date of birth or age; 4) Subject sex; 5) SAE (verbatim term); 6) SAE onset; 7) Brief description of event, course, and countermeasures taken; 8) Relatedness; 9) Intensity; 10) Seriousness; 11) Outcome; and 12) Relevant history/pre-existing medical conditions.

All additional information concerning the SAE until trial termination or definite outcome should be communicated per follow-up report without delay. The immediate and follow-up reports must only identify the subjects using the unique subject identifier. The investigator is obligated to comply with applicable regulatory requirement(s) related to the reporting of SAE to the regulatory authorities and the relevant IRB.

8.5 Stopping Rules

If SAEs occur that are not tolerable, the investigator will decide for that subject whether to stop the trial and/or treatment of the subject. This study may be prematurely terminated, if after review by the DSMB, there is sufficient reasonable cause. Written notification documenting the reason for study termination will be provided to the investigators and the OHSU Coordinating Center. Circumstances that may warrant termination include, but are not limited to: 1) Determination of unexpected, significant, or unacceptable risk to patients; 2) Inadequate enrollment with failure to enter patients at an acceptable rate; 3) Insufficient adherence to protocol requirements; and 4) Insufficient complete and/or evaluable data. This study may be discontinued at any time by the DSMB’s and or responsible IRB’s recommendation.

9 STUDY OVERSIGHT

9.1 Quality System

The OHSU Coordinating Center is responsible for implementing and maintaining quality assurance and quality control systems with a written manual of operating procedures and standard operating procedures.

9.2 Promoting and Monitoring Adherence to the Research Protocol

Adherence to protocol is important since it is impossible to blind caregivers to the study regimens. The research coordinator will monitor compliance to the study protocol by auditing instructions given to comply with the study protocol. We will conduct 100% audits of the protocol instructions in the first 5 patients per site, and with random checks subsequently, to monitor protocol compliance. All study personnel will undergo extensive training in the conduct of the protocol, the measurement of vascular pressure, and the neurological exam for ASIA scoring.

An academic detailing process will be used to educate practitioners about the use of guidelines and the procedures to correctly implement the study protocol, the importance of the clinical goals and the achievement of the established targets, and the importance of the study in improving the knowledge of the short and long term effects of blood pressure management. Maintenance of nurses and physicians adherence to the study protocol will be enhanced by quarterly meetings with sites PIs (phone calls) to facilitate discussion and stress the importance of compliance. The meetings will also provide an opportunity to discuss deviation from protocol in specific instances.

Additional strategies include minimizing the complexity of the treatment regimen, maximizing the therapeutic relationship between study staff, clinical staff and subjects (e.g., frequent friendly contacts such as telephone calls, newsletters, post cards, birthday cards and limiting the number of staff who work directly with subjects), providing sufficient information/education, ensuring important communication with subjects is simple, direct and repeated, making it convenient for subjects to participate, and involving family or other social supports in the process.

9.3 Monitoring of Study Conduct

The study will be monitored in accordance with current GCPs. The Clinical Research Associates at the Coordinating Center will be in contact with the participating sites to ensure that the study is being conducted in accordance with the protocol and that AE data is entered in a timely fashion. Audits of compliance with protocol instructions will be carried out intensively especially at the beginning of the trial to ensure complete understanding of such instructions. AE data entered on the CRFs will be reviewed by the Research Monitor as indicated above. The DSMB will be notified of any serious events, deaths, unexpected events or potential trends in accordance with the data safety plan. The site investigators will grant permission to the OHSU Coordinating Center or authorized designee, and appropriate regulatory authorities to conduct on-site monitoring of all

appropriate study documentation. When necessary, the monitoring visits will be conducted in poor performing sites due to poor enrollment, incomplete data entry, or any other potential problem with study conduct, to promote collection of high data quality and preserve the integrity of the study, and also further ensure patient safety in the event that unreported events or other unintentional study misconduct is occurring. Protocols exceptions and violations will be scrutinized on a case-by-case basis, and exceptions granted if appropriate. Queries and data clarifications requests will be also generated by the data and biostatistical group to assist in monitoring the conduct of the study and ensure high data quality.

9.4 Study Monitoring and Site Visits

Trial site monitoring will be performed by OHSU personnel on an as-needed basis depending on the progress of the trial. The authorized delegates, applicable SOPs, the frequency of monitoring visits, and the reporting modus, will be defined in writing as required by sponsor SOPs. Monitored sites will be informed about visit outcomes using a follow-up letter.

Monitoring and auditing procedures developed by OHSU Coordinating Center or its designee will be followed, in order to comply with GCP/ICH guidelines. When needed, on-site checking of the database for completeness, clarity, and consistency as compared with source documents and clarification of administrative matters will be performed. Monitoring will be done by personal visits from a representative of OHSU Coordinating Center (site monitor) who will review the source documents. The site monitor will ensure that the investigation is conducted according to protocol design and regulatory requirements by frequent communications (letter, telephone, fax, etc.).

The site investigators will grant permission to the OHSU PI or authorized designee, and appropriate regulatory authorities to conduct on-site monitoring of all appropriate study documentation. Each site will have an initiation visit and will be monitored yearly if more than 10 patients have been enrolled in the study. A close out study visit will be carried out when all the patients have completed the study. Protocols exceptions and violations will be scrutinized on a case-by-case basis, and exceptions granted if appropriate. Queries and data clarifications requests will be also generated by the data and biostatistical group to assist in monitoring the conduct of the study and ensure high data quality. Corrections, amendments, or clarifying statements resulting from the monitoring visit will be made by the investigator where necessary.

9.5 Adverse Event Monitoring

The Research Monitor will remain blinded to treatment assignment and will review listings of AEs regularly throughout the trial to assess for trends or unexpected events. If potential trends or unexpected events are noted, a summary will be provided to the DSMB for further review and evaluation. In the case of a SAE (as defined in 21 CFR 312.32) the investigators will notify the DSMB chair in a timely manner according to the agreed schedule. A complete report documenting the AE and including a description of the event, signs and symptoms, severity, any interventions initiated, the duration and the outcome, as well as the probable cause will be generated. The

study PIs will provide a written notification of reportable events to the funding agency in accordance with federal regulations. All SAEs will be followed until resolution or stabilization.

9.6 Research Monitor

The Research Monitor is responsible to oversee the safety of the research and report observations/findings to the IRB or a designated institutional official. The Research Monitor will review all unanticipated problems involving risks to subjects or others associated with the protocol and provide an independent report of the event to the IRB. The Research Monitor may discuss the research protocol with the investigators; shall have authority to stop a research protocol in progress, remove individual human subjects from a research protocol, and take whatever steps are necessary to protect the safety and well-being of human subjects until the IRB can assess the monitor's report; and shall have the responsibility to promptly report their observations and findings to the IRB or other designated official and the HRPO.

9.7 Data Safety and Monitoring Board

Safety will be monitored on an ongoing basis throughout the trial by a DSMB appointed by OHSU Coordinating Center. The DSMB will include an orthopedic surgeon, an epidemiologist, and a biostatistician.

Reports providing a comprehensive overview of enrollment information, safety summaries (AE and SAE tabulations), and other pertinent data will be generated for review by the DSMB at agreed upon intervals. However, SAEs related to study treatments and any deaths will be reported immediately to the DSMB.

The DSMB will not be blinded to interim data, and will evaluate pre-specified safety endpoints in all interim analyses. Efficacy data will be reviewed, but no formal efficacy analyses will be conducted. The committee will meet before the trial begins to review the study protocol and the DSMB charter. The DSMB will meet at intervals via teleconference or in person as pre-specified in the DSMB Charter, with the first teleconference taking place about six months after the first patient has been enrolled to review the interim safety data to determine if the trial should stop for safety. An in-person meeting will occur after 50% of the study participants have completed the follow up. A final DSMB meeting will take place 6 months after the last patient completes follow-up. In addition to scheduled meetings to review interim results, the DSMB will meet as needed to review safety concerns raised by investigators.

The DSMB will review safety data, enrollment data, protocol violations, rates of treatment non-compliance, and overall study progress. Interim safety reports for the DSMB will be generated prior to each meeting or as requested by the DSMB. The DSMB can recommend early stopping of study or of any of the study arms for concerns of participant safety at any time during the study. The DSMB will decide, based on the committee members' judgment, what constitutes a significant

safety concern that would potentially lead to stopping the trial early. There are no priori stopping rules for efficacy.

9.8 Steering Committee

A Steering Committee will have oversight on site performance. There will be an initial planning meeting and the members will meet regularly after the DSMB meeting. To maintain site enthusiasm and momentum with patient enrollment, a quarterly study update newsletter will also be distributed. There will be several attempts to remediate poor performing sites in terms of enrollment rates or study conduct, including on-site monitoring visits. If these issues cannot be corrected or if there are patient safety issues, further enrollment at that site will be terminated and the site replaced with one of the back-up sites.

9.9 Audits

Audits as defined by GCP will be performed for this trial. Regulatory authorities, the IRB, and/or the quality assurance group of OHSU Coordinating Center or its designee may request access to all source documents, printouts of the CRFs, and other study documentation for on-site audit or inspection. Direct access to these documents must be guaranteed by the investigator, who must provide support at all times for these activities. The investigator will permit the OHSU Coordinating Center personnel or authorized delegates to audit the trial facilities and documentation at agreed times.

9.10 Inspections

The investigator and/or personnel at other establishments are obliged to cooperate with any inspection of the documents, facilities, records, and other resources deemed appropriate by the inspecting authorities to be related to the trial and that may be located at the trial site, OHSU Coordinating Center or at other establishments. The investigator or personnel at other establishments should notify the OHSU Coordinating Center as soon as possible about any upcoming regulatory authority inspection.

10 STATISTICAL CONSIDERATIONS

10.1 Assessment of Study Variables

10.1.1 Primary Endpoint

The study primary endpoint is the average change in the motor and sensory ASIA Impairment Scale (AIS) scores at six months from baseline. The AIS score will be determined in accordance to the recommendation issued by the International Standards for Neurological Classification of SCI. The structured evaluation includes five steps for the determination of the sensory and motor level, the neurological level of injury and the completeness of the injury. Each step is described in detail below:

Step 1 determines the sensory level as the most caudal intact dermatome on the right and left sides. The best score with normal pin prick (PP) sensation will be considered the sensory level. The sensory sub-score will be used as primary sensory endpoint, defined as the sum the PP score on the right and left side. The maximum sensory score for both right and left sides is 112.

Step 2 determines the motor level as the most caudal key muscle group that has a strength grade of at least 3. For regions where there is no testable myotome, the motor level is presumed to be at the same level as the sensory one. The motor sub-score will be used as primary motor endpoint, defined as the sum the upper and lower extremities scores on the right and left side. The maximum score for both right and left sides is 100, however, due to our inclusion criteria, the maximum motor score at baseline will be 50.

Step 3 determines the neurological level of injury which is defined as the most caudal segment of the cord with intact sensation and antigravity muscle strength (at least strength of 3), e.g. the most cephalad of the sensory and motor levels determined in Step 1 and 2. Zone of partial preservation will also be noted.

Step 4 determines AIS Grade. For this study, only AIS grades A and B will be considered, though patients could progress and improve to grades C or higher. To determine whether the injury is complete (Grade A) a patient needs to have lost voluntary anal sphincter contraction and all S4-5 sensory scores and deep anal pressure. Grade B is defined as, "sensory but not motor function preserved below the neurological level and includes the sacral segments S4-5 (light touch or PP at S4-5 or deep anal pressure) AND no motor function is preserved more than three levels below the motor level on either side of the body."

10.1.2 Secondary Endpoints for Efficacy

In addition to the neurological exam, secondary endpoints will include measures of functional status and quality of life. The endpoints selected emphasize the ability to detect clinically

meaningful differences between study groups and to facilitate comparison of these study results to published studies from around the world. The following measured secondary endpoints will be assessed:

The International Spinal Cord Injury Basic Pain Data Set (ISCIBPDS) is a self-reported instrument that has been found to have high internal consistency and validity for 23 of the 27 pain interference items and scales. According to the guidelines for clinical assessment following acute cervical SCI, the ISCIBPDS is recommended as level I evidence as the preferred means to assess pain and its severity, physical and emotional functioning in SCI patients.

The Spinal Cord Independence Measure (SCIM III) is a disability scale developed specifically for patients with SCI that measures basic functional independence and accounts for functional impairments associated with tetraplegia or quadriplegia. The SCIM III is comprised of 19 items in three sub-scales including: 1) Self-care (6 items, sub-score 0-20); 2) Respiration and sphincter management (4 items, sub-score 0-40); and 3) Mobility (9 items, 0-40), which is subdivided into “room and toilet” and “indoors and outdoors”. The items are weighted in terms of their clinical relevance and the total score ranges from 0 to 100. To ensure consistency, the SCIM III will be scored by the study personnel via observation and interview.

The International Spinal Cord Injury Quality of Life Basic Data Set (ISCIQOL) is a brief 3-item instrument that assesses the general quality of life, and physical and psychological health satisfaction with life. The variables are rated on a scale ranging from 0 (completely dissatisfied) to 10 (completely satisfied). This instrument was developed by a committee of experts for the inclusion in the International Spinal Cord Injury Basic Data Set.

Data will also be collected on the following instruments:

- Activities and Participation
- Cardiovascular Function
- Pulmonary Function

The primary and secondary endpoints will be measured during the hospitalization and at the 6-month follow up, as appropriate.

10.1.3 Secondary Endpoints for Safety

Participants will be monitored for complications throughout the trial. Safety data will be collected during the study period to determine the safety profile of TPM. Safety will be addressed by assessing AEs, blood pressure, HR, and urine output. Routine clinical safety laboratory testing includes blood chemistry and hematology values that contribute to the computation of the organ dysfunction score, as described below. The following complications will be prospectively measured and evaluated.

Respiratory complications: Measured by a composite score of acute respiratory distress syndrome, hypoxemia as indicated by PaO_2/FiO_2 ratio, pulmonary edema requiring diuretics, and pneumonia;

Occurrence of myocardial ischemia or infarction: Measured by troponin levels;

Maximum organ dysfunction: Measured by the sequential multiple organ failure score (SOFA) in the first seven days or until ICU discharge after injury. The SOFA score is made of six variables, each representing an organ system. Each organ system is assigned a point value from 0 (normal) to 4 (high degree of dysfunction/failure), with a range from 0 to 24. Components of the SOFA score include PaO_2/FiO_2 ratio, Glasgow Coma Scale, MAP or administration of vasopressors, bilirubin, platelets, and creatinine. All components of the SOFA score will be collected daily for seven days or until ICU discharge, by selecting the worst physiological value. The worst measurement is defined as the measure that yields the highest number of points. When components (laboratory values) are not measured, they will be considered missing (9 points assigned);

Organ System	0	1	2	3	4	9
**Respiratory** PaO_2 / FiO_2 (mmHg)	>400	301-400	201-300 (with or w/o respiratory support[1]) <201 (w/o respiratory support[1])	101-200 (with respiratory support[1])	≤100 (with respiratory support[1])	Not measured
**Hematologic** Coagulation Platelets (x 10^9/L)	>150	101-150	51-100	21-50	≤20	Not measured
**Hepatic** Bilirubin mg/dl (or µmol/L)	<1.2<br><20	1.2 – 1.9<br>20-32	2.0-5.9<br>33-101	6.0-11.9<br>102-204	≥12.0<br>>204	Not measured
**Cardiovascular** Hypotension	MAP≥70 mmHg	MAP<70 mmHg	Dopamine≤5.0 (µg/kg/min) or any dose of: Dobutamine, Milrinone, Levosimendan	Dopamine 5.1-15.0 (µg/kg/min) or Epinephrine≤0.1 or Norepinephrine≤0.1[2]	Dopamine >15.0 (µg/kg/min) or Epinephrine>0.1 or Norepinephrine>0.1[2]	Not measured
**Neurologic** Glasgow Coma Score	15	13-14	10-12	7-9	≤6	Not measured
**Renal** creatinine mg/dl (or µmol/L) urine output	< 1.2<br>< 110	1.2-1.9<br>110-170	2.0-3.4<br>171-299	3.5-4.9<br>300-440<br>or <500 ml/day	≥5.0<br>>440<br>or <200 ml/day	Not measured

[1]Respiratory support is defined as any form of invasive or noninvasive ventilation including mask CPAP or CPAP delivered through a tracheostomy/tracheotomy or endotracheal tube.

[2]Or any dose of phenylephrine, vasopressin or metaraminol.

Duration of mechanical ventilation and hospital stay, and discharge disposition: Defined as any form of invasive or noninvasive ventilation including mask CPAP or CPAP delivered through a tracheostomy/tracheotomy or endotracheal tube.

10.2 Sample Size Estimation and Statistical Power

For the study's primary aim to compare the change in the 6-month ASIA scores (change from baseline) between the ABP and the CBP groups, a total of 126 patients will be needed to participate in the study to have statistical power of 80 percent to detect a 5-point mean between the two treatment groups. We assume a 10 point standard deviation in the ASIA measurements, a modest correlation between the baseline and six-month ASIA scores of 0.50, using a 2-sided five percent alpha-level test. To account for some data loss to follow-up, the sample size will be increased to 152. We chose a 5-point difference in the mean change score between the two treatment groups as a conservative effect size because the magnitude is considered a clinically meaningful difference in neurological recovery. Additionally, our calculations assume that the correlation between the pre-randomization ASIA scores and the 6-month ASIA scores is only modest ($r \geq 0.50$). For higher levels of correlation between the two scores, the statistical power well exceeds 80 percent. See Figure below.

Figure: Minimum effect sizes (ES) changes in ASIA scores between the treatment groups. These estimates assume a statistical power of 80 percent, sample sizes of n=63 per treatment group and a 2-sided, five percent alpha-level test. The correlations (horizontal axis) indicate the degree of correlation between the baseline and 6-month ASIA scores.

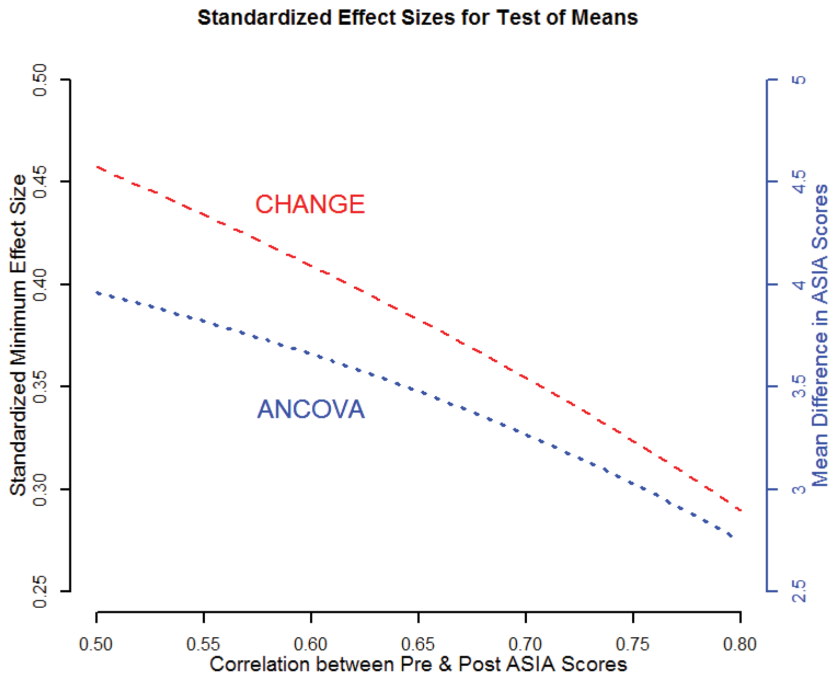

10.3 Statistical Analysis Plan

An intent-to-treat analysis will be performed. The primary outcome is the mean six-month ASIA motor function test score, comparing the ABP group to the CBP group. The primary analysis method will be linear regression. We will use robust (sandwich) standard error estimates in for test statistics and confidence intervals to remedy the possible minor violations of modeling assumptions (e.g., variance heterogeneity) in the six-month ASIA scores. The statistical model used for this analysis will be:

$$E[Y_1 \mid X, Y_0] = \beta_0 + \beta_1 X + \beta_0 Y_0$$

Where $E[Y_1]$ is the mean six-month ASIA score, X an indicator of the treatment assignment (1 = ABP, 0 = CBP), Y_0 is the pre-randomization (baseline) ASIA score, and (β_0, β_1, β_2) are the model parameters that are to be estimated from the patient data. This regression model is often referred to as an analysis of covariance (ANCOVA) model because we control for between patient variance in the baseline ASIA scores by including the baseline ASIA scores as a concomitant variable, in our treatment comparisons of the mean six-month ASIA scores. The estimates model parameter for the treatment assignment, β_1, can be represented as

$$\beta_1 = E[Y_1 \mid X = 1, Y_0] - E[Y_1 \mid X = 0, Y_0],$$

is interpreted as the difference in the mean six-month ASIA motor function test score for the ABP and CBP treatment groups, given that the patients being compared have the same baseline ASIA scores. In randomized trials β_1 is often interpreted as the mean *change* (from baseline) because the average baseline ASIA scores should be approximately equal in the two treatment groups. We chose to model the effect of treatment using analysis of covariance instead of comparing the intra-patient differences between the groups because the ANCOVA model yields a more precise test. The Figure above illustrates that the variance from the ANCOVA model us uniformly lower than the CHANGE model for varying levels of correlation between the baseline and 6-month ASIA scores.

For the analysis of quantitative secondary endpoints (i.e., SCIM III, ISCIBPDS, ISCIQOL, hospital length of stay, etc.) where the desired statistical summary is the comparison of mean changes between the treatment groups, we will employ the same linear regression strategy as the primary endpoint above. For binary (e.g., occurrence of myocardial ischemia or infarction) we will analyze the data using a logistic regression model

$$\log[odds(X)] = \beta_0 + \beta_1 X$$

where the $odds(X) = \pi(X) / [1\text{-}\pi(X)]$ and $\pi(X)$ is the probability of a given event. Here, the model parameter for the treatment assignment, $\beta_1 = \log[odds(\text{ABP})/\ odds(\text{CBP})]$ and $\exp(\beta_1)$ is the odds ratio (OR) comparing the odds of the event for the ABP group to the CBP group.

Finally, we will descriptively present baseline characteristics (e.g., age, gender, etc.) and safety endpoints (e.g., respiratory complications, SOFA scores, duration of mechanical ventilation, discharge disposition, etc.) in tabular format using means (SD) for quantitative characteristics and frequencies (with percentages) for categorical characteristics, in aggregate and stratified by treatment group.

11 DATA HANDLING AND RECORD KEEPING

11.1 Data Management Responsibilities

This study will use a combination of paper-based and electronic data capture. All study personnel doing data collection will be carefully trained in the task. The Manual of Operations/Data Dictionary will contain detailed instructions for each variable. The study nurses/coordinators and outcome examiners will have separate but parallel systems for ensuring that data are collected consistently and accurately.

The main study database will be maintained on a secure server cloud with established provisions delineated in the information system security plan. The data security plan prepared by the within the Data Management Unit (DMU) addresses confidentiality, security measures, back-ups, and other aspects of the operational status. Data encryption and authentication methods will be used. Data will be entered at the individual sites and uploaded on a regular basis. Data from the central reader will also be uploaded regularly. Data collection screens/forms will be designed so that questions are unambiguous and require a response. The use of text fields will be minimized. Language will be kept as simple as possible, using standard terminology.

11.2 Source Data

Source data is defined by GCP as “all information in original records and certified copies of original records of clinical findings, observations, or other activities in a clinical trial necessary for the reconstruction or evaluation of the trial. Source data are contained in source documents (original records or certified copies). Source data comprise clinical documentation, data, and records that describe or track the methods, conduct, or results of the trial, the factors affecting the trial, and the actions taken. All source data arising from the trial will be kept by the investigator, who must provide direct access for trial-related monitoring, audits, ethics committee review, and regulatory inspection. The nature and location of all source data will be identified and documented by the investigator to ensure that all sources of original data required to complete CRFs are known to the OHSU Coordinating Center and/or trial site personnel and are accessible for verification during trial-related monitoring, audits, relevant IRB review, and inspections.

11.3 Case Report Forms

This trial will utilize a combination of paper-based case report forms and electronic case report forms (eCRFs) with data capture, coding, and cleaning provided by the DMU. CRFs for each subject will be provided to the investigator by the coordinating center in paper and electronic format to document trial data. The investigator and personnel to whom this task is delegated will use the CRFs to record information required by the protocol. The investigator will verify and confirm by signature that the CRFs have been checked and are complete, accurate, and compatible with source documents. All CRF entries, corrections, and alterations will be made by the investigator or other authorized personnel under their supervision. Entries will be checked against appropriate source documents by the OHSU Coordinating Center.

11.4 Identifiers

Consenting participants will be assigned a unique study ID. This ID will be constructed from the site number (10, 11, 12, 13, 14, 15) and a unique randomization number for each site participant (001, 002, 003…).

The OHSU Coordinating Center will provide each clinical site with a list of valid study IDs. Clinical sites will maintain the only link between the participant ID and the actual participant identity. Unless local regulation stipulates otherwise, this link will be maintained on paper filed in locked cabinets at the clinical site (Participant ID Log). The security of this documentation will be verified at on-site monitoring visits.

11.5 Confidentiality

The DMU security policy provides data confidentiality and maintains data integrity while providing appropriate access. In addition, we have built extensive redundancy into our systems. The DMU provides access control and authentication in four domains: 1) physical security, 2) network access, 3) database access, and 4) data transmission. All data are collected on paper forms and entered into the password-protected database at each site. Only trained study personnel are allowed access to the database and the identity of the person entering or editing the data is tracked. Local databases are stored on password-protected computers that are backed up nightly. The databases are encrypted and uploaded weekly to the DMU. The DMU incorporates the new or modified data into the study master database. The DMU is located in a secure building in a room that is locked when it is unoccupied. Access to the computer, computer system, network drive and database are all password-controlled. The master database is kept on a secure server under conditions identical to that used for the hospital electronic medical records. This includes access by key-card and regularly scheduled backup.

Except for dates, study data will contain no personally identifying information (e.g., name, social security number, address, telephone numbers, email address, etc.). Identifiable data (associated with a study assigned ID) will be retained only on participants who have signed informed consent. The consent will contain a statement on confidentiality explaining how the data will be safeguarded and who will or might have access to the data, including representatives from the Department of Defense as required by the Program Announcement.

Clinical sites will be required to maintain study files on each consenting participant in order to provide source documentation for the data collected. These files will be secured according to local requirements for medical records since they will contain personally identifying information. Where the clinical site has completely migrated to an electronic medical records (EMR) system, the study staff at the site will need to give EMR access to study monitors or provide printed copies of relevant source documentation for onsite monitoring visits. Printed copies of relevant source documentation will be destroyed following the monitoring visit per local regulation for medical records.

All data will be entered electronically at the clinical site by trained, authorized study staff using the web based system. Access to the eCRF system requires knowledge of an individually assigned userID and password. Passwords are required to meet certain strength levels and to be changed every 90 days. All activity (entering, updating data and responding to queries) is logged electronically with the userID of the person entering or making the change to the data. eClinical OS uses a group security model. Clinical site study staff will be able to add patients, enter, review and modify data and respond to data queries regarding participants enrolled at their site.

Study coordinating center staff who have a need to review study data online will have read-only access to the eCRF system. Study data coordinators and monitors will have read-only access to the study data and will be able to post, review and close queries. Statisticians will have the capability to download study data and randomization data for analysis. Each staff member will be trained on the use of the system and will be assigned an individual userID.

11.6 Data Entry Completion

OHSU Coordinating Center or its designee will instruct the study center regarding data capture procedures on electronic and/or paper CRFs. It is the investigator’s responsibility to ensure the accuracy, completeness, and timeliness of the data reported for each patient. Source documentation supporting the data should indicate the patient’s participation in the study and should document the dates and details of study procedures, AEs, and patient status. The investigator, or designated representative, should complete data entry as soon as possible after information is collected, preferably on the same day that a study patient is seen for an examination, treatment, or any other study procedure. Any outstanding entries must be completed immediately after the final examination. An explanation should be given for all missing data. The investigator must sign and date the Investigator’s Statement that will be supplied to endorse the recorded data.

11.7 Study Records Retention

The investigator is responsible for the filing of all essential documents in an investigator’s site file and the OHSU Coordinating Center is responsible for the timely filing of all essential documents in the trial master file. As applicable, these files must be available at monitoring visits and during audits or regulatory inspections. After trial completion, the investigator will ensure that all source data/documentation related to the trial is recorded, handled, and stored in a way that allows its accurate reporting, interpretation, and verification. The investigator will take measures to prevent accidental or premature destruction of these documents. The investigator will keep the investigator’s site file, the source data/documentation related to the trial according to the prescribed record retention period according to the hospital policy, or at least until informed by the OHSU Coordinating Center that the trial-related records are no longer required.

11.8 Data and Resources Sharing Plan

Because a significant amount of the support for this study is being possibly provided by the Department of Defense (DoD), the DoD will have access to both the identified and the de-identified data. It is the intent of the investigators to share a copy of the final study database to the funding Institution. Primary interest in these data will be from the trauma, orthopedics, neurosurgery, and critical care community. Further, the Department of Defense has the infrastructure to ensure that the data will be used only for legitimate purposes. The trial database will be de-identified in accordance with HIPAA privacy requirements and will be delivered no sooner than publication of the primary results paper from this clinical trial. Details of the data sharing plans will be discussed with the program official and verified for compliance with institutional policies, IRB rules as well as local, state and Federal regulations.

11.9 Data Quality Assurance

The accuracy and reliability of the trial data will be assured by the performance of a combination of trial site visits, training, monitoring visits, data cleaning, and audits.

12 PUBLICATION/DATA SHARING POLICY

It is anticipated that the results of this study will be presented at scientific meetings and/or published in a peer-reviewed scientific or medical journal. A Publications and Presentation Committee, comprised of investigators participating in the study and representatives of the steering committee, as appropriate, will be formed to oversee the publication of the study results, which will reflect the experience of all participating study centers. Subsequently, individual investigators may publish results from the study in compliance with their agreement with OHSU Coordinating Center. A pre-publication manuscript is to be provided to the funding agency when accepted by the publisher.

13 LITERATURE REFERENCES

1. Ryken TC, Hurlbert RJ, Hadley MN, et al. The acute cardiopulmonary management of patients with cervical spinal cord injuries. *Neurosurgery.* 2013;72 Suppl 2:84-92.
2. Hurlbert RJ, Hadley MN, Walters BC, et al. Pharmacological therapy for acute spinal cord injury. *Neurosurgery.* 2013;72 Suppl 2:93-105.
3. Levi L, Wolf A, Belzberg H. Hemodynamic parameters in patients with acute cervical cord trauma: description, intervention, and prediction of outcome. *Neurosurgery.* 1993;33(6):1007-1016; discussion 1016-1007.
4. Vale FL, Burns J, Jackson AB, Hadley MN. Combined medical and surgical treatment after acute spinal cord injury: results of a prospective pilot study to assess the merits of aggressive medical resuscitation and blood pressure management. *J Neurosurg.* 1997;87(2):239-246.
5. Communal C, Singh K, Pimentel DR, Colucci WS. Norepinephrine stimulates apoptosis in adult rat ventricular myocytes by activation of the beta-adrenergic pathway. *Circulation.* 1998;98(13):1329-1334.
6. Schoenfeld AJ, Laughlin MD, McCriskin BJ, Bader JO, Waterman BR, Belmont PJ, Jr. Spinal injuries in United States military personnel deployed to Iraq and Afghanistan: an epidemiological investigation involving 7877 combat casualties from 2005 to 2009. *Spine (Phila Pa 1976).* 2013;38(20):1770-1778.

APPENDIX A: SCHEDULE OF EVENTS

Visit Description	Pre-randomization	Treatment Phase							Discharge	Follow-Up
Study Visits/ Study Days (or weeks)	ICU Day 0	ICU Day 1[a]	ICU Day 2	ICU Day 3	ICU Day 4	ICU Day 5	ICU Day 6	ICU Day 7	Hospital Discharge	6 Month f/u
Inclusion/Exclusion	X									
Informed Consent	X									
Verbal Consent and Continued Participation Form *(if applicable)*	X	X	X	X	X	X	X	X	X	
Demographics	X									
Injuries and Past Medical History	X[b]									
Injury Assessment *(date/time of symptom onset; mechanism of injury)*	X									
Injury Severity Score	X									
ASIA Assessment	X			X[c]						X
Spine Imaging Data	X									
Spine Procedure Data	X									
Randomization	X									
Targeted blood Pressure Management	X	X	X	X	X	X	X	X		
BP Non-Compliance	X	X	X	X	X	X	X	X		

Vital Signs	X	X[d]	X[d]	X[d]	X[d]	X[d]	X[d]	X[d]		
Mechanical Ventilation	X	X	X	X	X	X	X	X	X	X
Fluid Intake/Output	X	X	X	X	X	X	X	X		
SOFA Assessment		X	X	X	X	X	X	X		
Vasopressor Log		X	X	X	X	X	X	X		
Concomitant Medications		X	X	X	X	X	X	X	X	X
Discharge Disposition									X	
SCI Basic Pain Dataset										X
SCI Independence Measure										X
Quality of Life										X
Hemodynamic Status										X
Serious Adverse Event Monitoring	X	X	X	X	X	X	X	X	X	X
Adverse Event Monitoring	X	X	X	X	X	X	X	X	X	X

ICU: Intensive Care Unit; SCI: Spinal Cord Injury; ASIA: American Spinal Injury Association; SOFA: Sequential Organ Failure Assessment.
a – ICU Day 1 begins at 0700 the day following randomization.
b – Medical history relevant to the trial and specific to hypertension will be documented.
c – ASIA assessment will be collected between 72 hours after injury and prior to ICU discharge.
d – Vitals and fluid intake/output will be collected every 4 hours while the patient is in the ICU.

APPENDIX B: INTERVENTION ALGORITHMS

ALGORITHM — Conventional blood pressure (CBP)

CONVENTIONAL BLOOD PRESSURE MANAGEMENT	
**Actual MAP <65**	**Norepinephrine or phenylephrine drip or other vasopressor**
**Actual MAP 65-70**	**Continue Current Care**

>> Measure and record MAP every 4 hours

>> On algorithm, select corresponding patient MAP interval

>> Follow instruction in corresponding colored cell

TEMPLE

Trial of Early Management of Pressure Following Acute Spinal Cord Injury

Patient Name/ID

Date: ___/___/___ Time: ___:___

☒ Document Non-Invasive Blood Pressure in the medical record every 4 hours

☒ Maintain MAP 65-70 mm Hg

If MAP <65 mm Hg

☐ Start **phenylephrine** infusion at 0.5 mcg/kg/min or _____________ mcg/kg/min and adjust rate by 0.1 mcg/kg/min or ___________ mcg/kg/min at 5 minute intervals, as needed.

☐ Start **norepinephrine** infusion at 0.02 mcg/kg/min or ___________ mcg/kg/min and double rate and adjust rate by 0.1 mcg/kg/min or ___________ mcg/kg/min at 5 minute intervals, as needed.

☐ Start **vasopressin** infusion at 0.4 IU/min, range 0.1-0.4 IU/min

___________________________ MD

___________________________ VORV

Research Coordinator: [Name]: Pager xxx-xxxx;
Principal Investigator: [Name]: Pager xxx-xxxx

CBP

ALGORITHM — Augmented blood pressure (ABP)

AUGMENTED BLOOD PRESSURE MANAGEMENT	
Actual MAP <85	Norepinephrine or phenylephrine drip or other vasopressor
Actual MAP 85-90	Continue Current Care

>> Measure and record MAP every 4 hours

>> On algorithm, select corresponding patient MAP interval

>> Follow instruction in corresponding colored cell

TEMPLE Study

Trial of Early Management of Pressure Following Spinal Cord Injury

Patient Name/ID

Date: ___/___/___ Time: ___:___

☒ Document Non-Invasive Blood Pressure in the medical record every 4 hours

☒ Maintain MAP 85-90 mm Hg

If MAP <85 mm Hg,

☐ Start **phenylephrine** infusion at 0.5 mcg/kg/min or _____________ mcg/kg/min and adjust rate by 0.1 mcg/kg/min or ___________ mcg/kg/min at 5 minute intervals, as needed.

☐ Start **norepinephrine** infusion at 0.02 mcg/kg/min or ___________ mcg/kg/min and double rate and adjust rate by 0.1 mcg/kg/min or ___________ mcg/kg/min at 5 minute intervals, as needed.

☐ Start **vasopressin** infusion at 0.4 IU/min, range 0.1-0.4 IU/min

_____________________________ MD

_____________________________ VORV

Research Coordinator: [Name]: Pager xxx-xxxx;
Principal Investigator: [Name]: Pager xxx-xxxx

ABP
