# Supplementary material for: Early Blood Pressure Targets in Acute Spinal Cord Injury: A Randomized Clinical Trial
Source: JAMA Netw Open. 2025 Sep 18;8(9):e2525364. doi: 10.1001/jamanetworkopen.2025.25364 (PMC12447234; doi:10.1001/jamanetworkopen.2025.25364)
Supplement: Supplement 3. — Data Sharing Statement [file jamanetwopen-e2525364-s003.pdf]

# Data Sharing Statement

Sajdeya. Early Blood Pressure Targets in Acute Spinal Cord Injury. *JAMA Netw Open*. Published September 18, 2025. doi:10.1001/jamanetworkopen.2025.25364

**Data**
**Additional Information:** NCT02878850
**Data available:** Yes
**Data types:** Deidentified participant data
**How to access data:** Data are available upon requests by contacting the senior author atmiriam.treggiari@duke.edu
**When available:** beginning date: 03-30-2026

**Supporting Documents**
**Document types:** Other (please specify)
**Additional Information:** Study Protocol
**How to access documents:** The full Study Protocol will be available as an electronic supplement and upon requests by contacting the corresponding author at miriam.treggiari@duke.edu
**When available:** With publication

**Additional Information**
**Who can access the data:** Data will be made available to researchers whose proposed use of the data has been approved.
**Types of analyses:** Data will be made available for analyses consistent with the approved request for use.
**Mechanisms of data availability:** Data will be made available with investigator support after approval of a proposal.
